# Wealth, income and HIV in sub‐Saharan Africa: a systematic review

**DOI:** 10.1002/jia2.70060

**Published:** 2025-12-23

**Authors:** Kaitlyn Atkins, Kirsty M. Sievwright, Holly Nishimura, Caitlin E. Kennedy

**Affiliations:** ^1^ Department of International Health Bloomberg School of Public Health Johns Hopkins University Baltimore Maryland USA; ^2^ HIV Center for Clinical and Behavioral Studies Division of Gender Sexuality and Health Columbia University/New York State Psychiatric Institute New York New York USA; ^3^ Department of Medicine Center for AIDS Prevention Studies University of California San Francisco San Francisco California USA

**Keywords:** wealth, income, inequality, socio‐economic status, systematic review, sub‐Saharan Africa

## Abstract

**Introduction:**

While economic vulnerability is an established driver of health disparities, the relationships between HIV and wealth, income, and economic inequality have been less consistently established. We conducted a systematic review of studies examining associations between wealth, income, and economic inequality and HIV incidence and prevalence in sub‐Saharan Africa (SSA).

**Methods:**

Following PRISMA guidelines, we searched PubMed, SCOPUS, Embase, EconLit and PsycINFO for quantitative publications through June 2024 examining the relationship between wealth, income or inequality and HIV status, acquisition, prevalence or incidence in SSA. From September 2022 to October 2024, we extracted data using standardized forms, assessed risk of bias and qualitatively summarized results.

**Results:**

Overall, 47 studies covering 48 countries met the inclusion criteria. Studies had generally low risk of bias, and most focused on a single country (*n* = 38), assessed household wealth as the exposure (*n* = 36) and employed cross‐sectional designs (*n* = 33). Studies assessing wealth and HIV incidence consistently identified a protective effect, while findings around HIV incidence and income were mixed. In studies assessing HIV prevalence, findings on HIV and individual and household income or wealth were mixed. Economic inequality was consistently associated with increased HIV prevalence at community, sub‐national and national levels.

**Discussion:**

Most included studies were cross‐sectional, among the general population, and secondary analyses of existing data. These can generate new insights about potential economic predictors of HIV, but longitudinal research is needed to understand economic impacts on HIV in evolving programme and policy contexts. Limited studies outside the general population highlighted opportunities for future research exploring economic drivers of HIV among the key population and potential differences in the HIV‐wealth relationship by gender and urbanicity.

**Conclusions:**

The evidence on HIV and wealth or income is mixed and varies by setting and population, while a limited literature suggests that economic inequality is more consistently associated with HIV risk. Longitudinal research is needed to assess causal relationships between economic factors and HIV, and to identify potential mediators of this relationship.

## 1 INTRODUCTION

Socio‐economic status (SES) has long been understood as a fundamental cause of health inequalities [1−4]. Several studies have documented a relationship between SES and HIV [5]. However, consensus on the SES‐HIV relationship in sub‐Saharan Africa (SSA)—home to 62% of people living with HIV [6] and where 34.9% live on < 2.15 U.S. dollars (USD) per day [7]—is limited [8].

**Figure 1:**
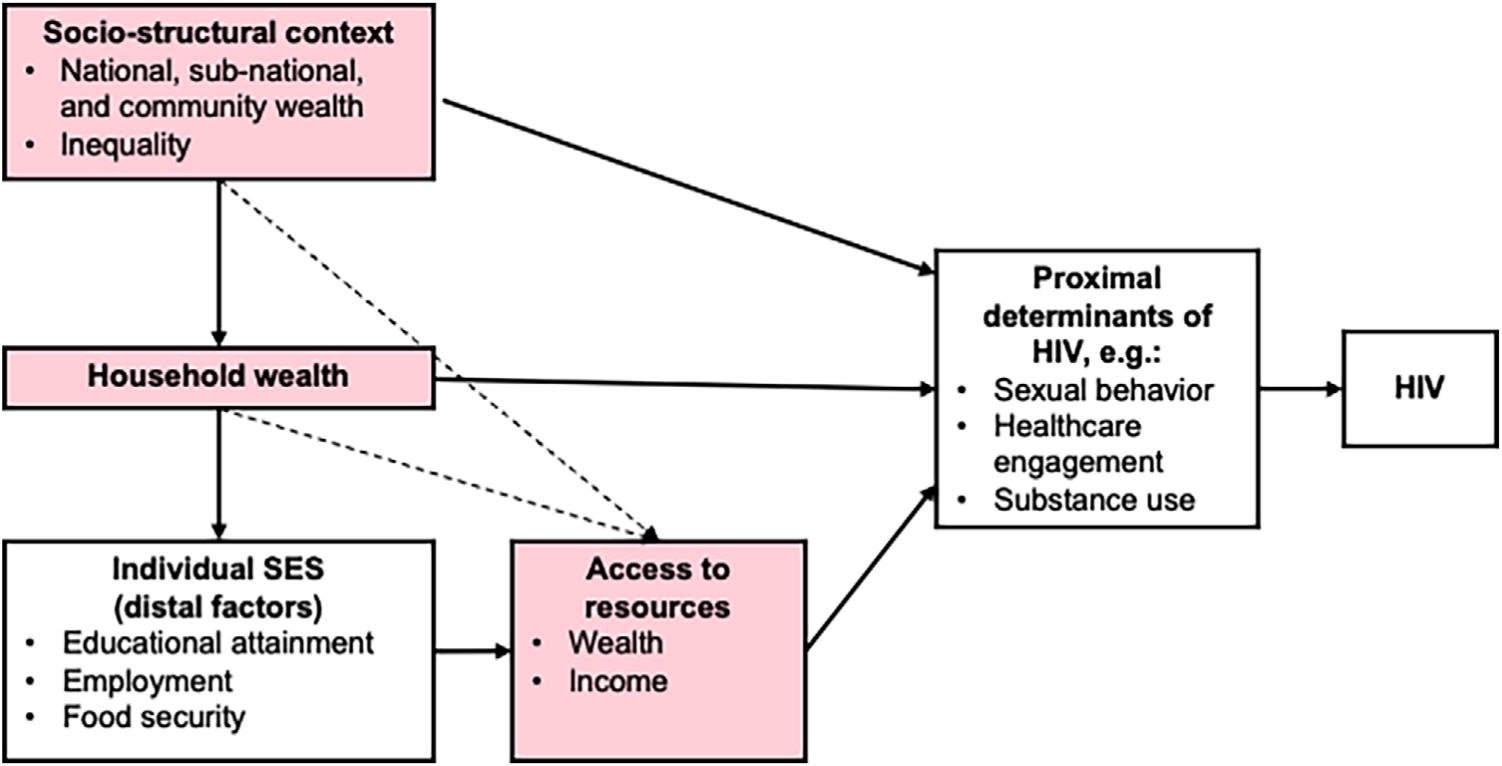
Conceptual model of socio‐economic status as a fundamental cause of HIV. *Note*: Shaded boxes represent factors assessed in the present review. Abbreviation: SES, socio‐economic status.

SES encompasses dimensions including education, occupation, wealth and income [9]. Fundamental cause theory [1] can be applied to consider SES a hierarchical structure in which material dimensions (e.g. wealth/income) represent economic resources through which upstream dimensions (e.g. education/occupation) shape health indirectly (Figure 1) [10, 11]. A growing body of evidence suggests the SES‐HIV relationship is more complex than previously conceptualized. Around the peak of the African HIV epidemic, Buvé et al. [12] identified how poverty facilitated the spread of HIV, in turn impoverishing communities through decreased economic productivity. Others have suggested that wealth, not poverty, may increase the risk for HIV in SSA [13−16], illustrated by a 2005 study in Tanzania [17], where HIV prevalence was four times higher among the wealthiest versus poorest women. Others note the SES‐HIV relationship may differ by gender, urbanicity or time [18−20], with emerging evidence indicating inequality may influence HIV incidence more than absolute wealth [8, 21, 22].

Despite uncertainty around the SES‐HIV relationship, economic strengthening interventions are increasingly used to improve HIV disparities [23]. Three 2018 reviews found that economic interventions were associated with improved HIV testing and care seeking [24−26]. However, the reviews found limited and underpowered evidence linking economic interventions to clinical outcomes, suggesting that economic initiatives alone may be insufficient to reduce HIV incidence [26]. The foundational assumption underlying these interventions is that economic resources reduce HIV risk in non‐experimental settings. However, observational literature around SES and HIV prevalence or incidence has not been well‐summarized, making assumptions difficult to check. Synthesizing this evidence is essential to guide interventions and support goals to end the HIV epidemic [6]. Existing reviews have focused on specific populations (e.g. women [21]) or behaviours (e.g. treatment adherence [27]), but to our knowledge, no review has thoroughly examined inequality as a driver of HIV in SSA [8]. We aimed to review and synthesize literature from observational studies characterizing relationships between wealth, income, inequality and HIV in SSA.

## 2 METHODS

Using Preferred Reporting Items for Systematic review and Meta‐Analyses (PRISMA) guidelines [28], we assessed associations between HIV incidence or prevalence and SES through measures of *wealth* (stock of assets or access to goods and services); *income* (household, individual or community earnings); and *inequality* (distribution of income or wealth between individuals, households or members of a community or state [29]).

### 2.1 Inclusion criteria

We required studies to quantitatively measure wealth, income or inequality; measure HIV prevalence or incidence; quantitatively and substantially assess the relationship between wealth/income and HIV prevalence or incidence; present data from SSA; and be published as a peer‐reviewed journal article. While studies directly measuring the impact of SES on incident HIV are preferable from a causal inference standpoint, very few studies in the literature prospectively assess this relationship. We, therefore, included cross‐sectional studies, while acknowledging the limitations of such analyses (e.g. potential for reverse causation).

Studies measuring only behavioural outcomes (e.g. transactional sex, condom use) were excluded. We further excluded qualitative studies; review papers, conference presentations or dissertations; studies that did not directly measure income, wealth or inequality; studies that did not substantially focus on the HIV‐income relationship; and studies assessing the impact of economic interventions, which have been explored elsewhere [26].

### 2.2 Search strategy and screening

In consultation with an expert health science informationist, we searched five electronic databases (PubMed, SCOPUS, Embase, EconLit and PsycINFO) from 1 January 2000 through 30 June 2024. Our search string included terms for HIV, income/wealth and SSA (Supporting Information).

We screened titles and abstracts of all citations; screening was completed in August 2024. We obtained full‐text articles of all selected abstracts, and two independent reviewers assessed articles for eligibility, with discrepancies resolved through consensus. We used forward and backward citation tracking to identify additional articles for screening.

### 2.3 Data extraction

Data were extracted independently and in duplicate using standardized forms, with differences resolved through consensus. We gathered the following:

- **Population**: Location; sample size; age; sex/gender; SES
- **Methods**: Study objectives; sampling approach; follow‐up periods and response rates; study design; exposure and outcome measures; analytic approach
- **Outcomes**: Effect sizes; confidence intervals; significance levels; conclusions; limitations

If measures of association were not reported in a study, we reached out to the authors to request this information. We assessed risk of bias (ROB) in each study using a modified version of the AXIS tool [30] (Supporting Information). The modified ROB tool included 10 items assessing rigour in study objectives, sampling, measures, statistical methods, presentation of results, interpretation of findings and ethics. Data extraction and ROB assessments were completed in October 2024.

### 2.4 Data analysis and synthesis

Where more than two studies used the same independent variable, we used meta‐analysis with random‐effects models to pool measures of association in Stata Version 18 (StataCorp: College Station, TX). Detailed methods are in the Supporting Information. We further synthesized findings based on exposure measures and coding categories. We categorized studies based on their exposures and level of analysis. When data were available, we conducted stratified analyses by gender and urbanicity.

## 3 RESULTS

### 3.1 Search results

**Figure 2:**
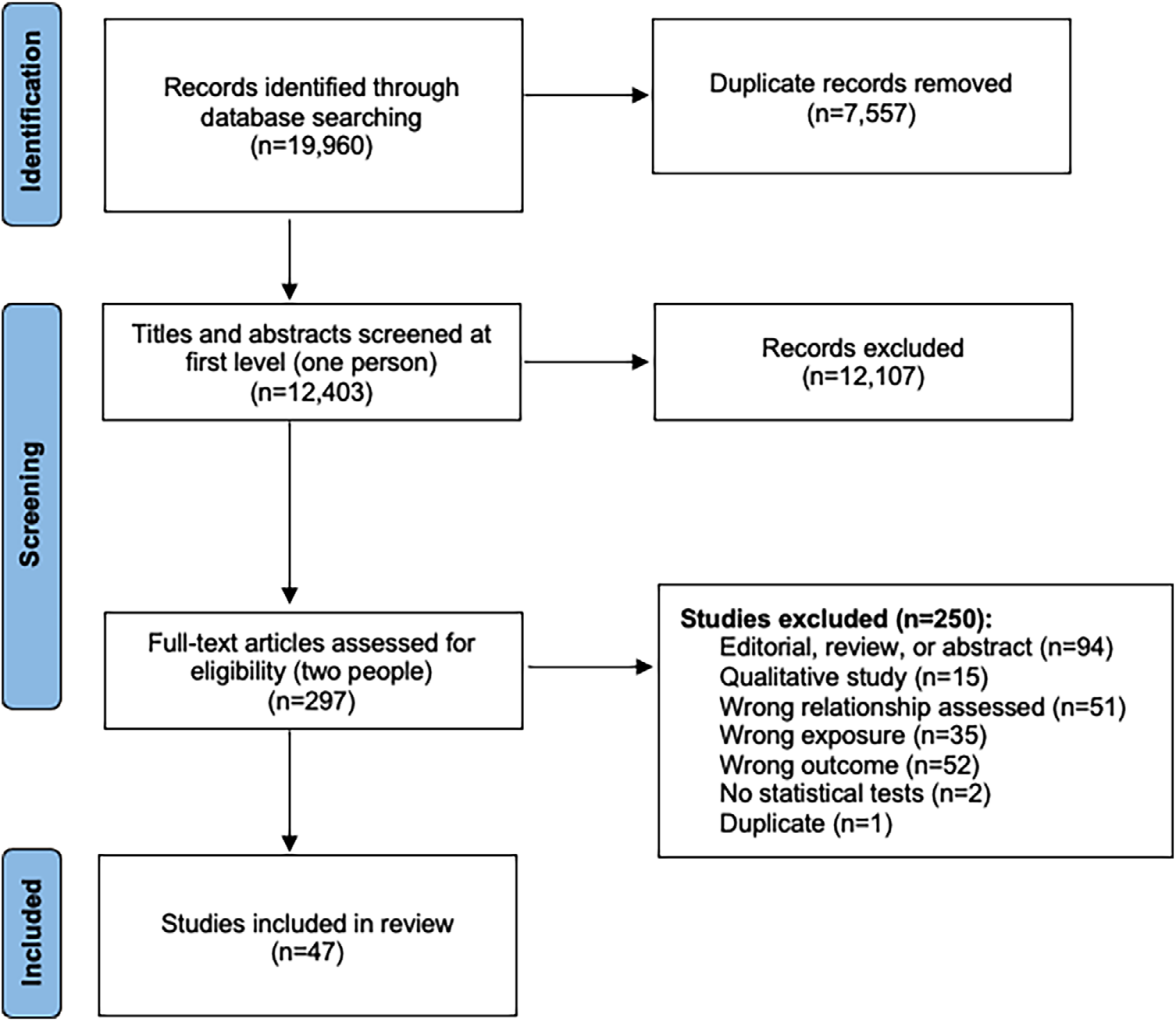
PRISMA flow chart representing disposition of citations through the search and screening process.

Our searches yielded 19,960 records, including 7557 duplicates, which were removed (Figure 2). A further 12,107 titles were removed during title and abstract screening. During full‐text screening, 250 were removed because they (1) were editorials, reviews or abstracts; (2) did not measure HIV outcomes of interest; or (3) did not assess the relationship between wealth/income and HIV. Ultimately, 47 studies were included in the review.

### 3.2 Description of included studies

Table 1 presents a summary of the 47 included studies [8, 19, 20, 31−74], covering 48 countries in SSA. Nine studies included data from multiple countries, while 35 focused on a single country. Tanzania (*n* = 11), Kenya (*n* = 10), Malawi (*n* = 10), South Africa (*n* = 10) and Zimbabwe (*n* = 10) were the countries most represented. Twenty‐nine studies used nationally representative data, while 18 used data from specific regions or cities.

**Table 1:** Description of included studies (*n* = 47)

First author, publication year, study design, socio‐ecological level(s) addressed	Population (*N*); study location(s); year(s)	Exposure(s)	Outcome (HIV incidence/prevalence)	Measures of wealth/income
**Longitudinal studies (all household levels)**				
Aulagnier, 2011, CS, HH	Women and men aged 12 and older (*n* = 3168); Windhoek, Namibia; 2006−2009	Wealth	Incidence	Household consumption versus poverty line
Barnighausen, 2007, CS, HH	Women aged 15−49 and men aged 15−54 (*n* = 3325); KwaZulu‐Natal, South Africa; 2003−2005	Wealth	Incidence	Household asset index
Gritzman, 2005, CS, HH	Women and men (age NR, *n* = 387 households); South Africa; 2011	Income	Incidence	Per capita income
Lopman, 2007, CS, HH	Women aged 15−44 and men aged 17−54 (*n* = 9454); Zimbabwe; 1998−2003	Wealth	Incidence	Household asset index
Nattrass, 2012, CS, HH	Black adolescents and young adults aged 14−22 (*n* = 4752); South Africa; 2002−2009	Wealth	Prevalence	Household asset index
Santelli, 2021, CS, HH	Women and men aged 15−49 (*n* = 43,600); Uganda; 1994−2018	Wealth	Incidence	Household asset index
Schur, 2015, CS, HH	Women and men aged 15−44 (*n* = 8201−13,335 households); Zimbabwe; 1998−2011	Wealth	Prevalence	Household asset index
**Case‐control studies (individual level)**				
Ogunmola, 2014, CC, I	Women and men aged 15−54 in a rural tertiary hospital (*n* = 237); Nigeria; Year NR	Income	Prevalence	Individual last‐month income
**Ecological studies (national level)**				
Ji, 2017, ES, N	General population (*n* = NR); 48 countries; 1985−2007	Income	Incidence	Per capita GDP
**Cross‐sectional studies, by socio‐ecological level**				
***Individual level***				
Mizinduko, 2020, XS, I	Female sex workers aged 18 and older (*n* = 958); Tanzania; 2017	Income	Prevalence	Individual income last sex
***Household level***				
Abimanyi‐Ochom, 2011, XS, HH	Women aged 15−49 (*n* = 19,021); Kenya, Uganda; 2003−2004	Wealth	Prevalence	DHS household wealth index
Andrus, 2021, RXS, HH	General population (*n* = NR); 27 countries; 2003−2016	Wealth	Prevalence	DHS household wealth index
Asiedu, 2012, XS, HH	Women aged 15−49 and men aged 15−59 (*n* = 31,712); Eswatini, Lesotho Malawi, Zimbabwe; 2004−2006	Wealth	Prevalence	DHS household wealth index
Bunyasi, 2017, XS, HH	Women aged 15−49 (*n* = 1906); Free State & Western Cape Provinces, South Africa; 2007−2008	Wealth	Prevalence	Household asset index
Bwana, 2023, XS, HH	Children under 5 (*n* = 576); Muehza District, Tanzania; 2015−2016	Wealth	Prevalence	Composite of household assets
Ekholuenetale, 2020, XS, HH	Women aged 15 and older (*n* = 4726); Mozambique; 2015	Wealth	Prevalence	DHS household wealth index
Ekholuenetale, 2021, XS, HH	Women aged 15−64 (*n* = 6501); Namibia; 2013	Wealth	Prevalence	DHS household wealth index
Emina, 2013, XS, HH	Women aged 15−49 (*n* = 6395); Malawi; 2010	Wealth	Prevalence	DHS household wealth index
Fortson, 2008, XS, HH	Women aged 15−49 and men aged 15−59 (*n* = 67,019); Burkina Faso, Cameroon, Ghana, Kenya, Tanzania; 2003−2004	Wealth	Prevalence	DHS household wealth index
Hadley, 2019, XS, HH	General population (*n* = 35,799 households); Ethiopia, Kenya, Tanzania; 2009 (Kenya) & 2011 (Ethiopia, Tanzania)	Wealth	Prevalence	DHS household wealth index, adjusted to reflect wage versus agricultural economy
Humphrey, 2008, XS, HH	Postnatal women aged 14 and older (*n* = 14,110); Zimbabwe; 1997−2000	Income	Prevalence	Self‐reported family income
Igulot, 2018, XS, HH	Women aged 15−49 and men aged 15−59 (*n* = 39,766); Uganda; 2004−2005, 2011	Wealth	Prevalence	DHS household wealth index
Ishida, 2012, RXS, HH	Women and men aged 15−49 (*n* = 17,552); Kenya; 2003, 2007	Wealth	Prevalence	DHS household wealth index
Kasirye, 2016, XS, HH	Women and men aged 15−49 (*n* = 19,534); Uganda; 2011	Wealth	Prevalence	DHS household wealth index
Lachaud, 2007, XS, HH	Women aged 15−49 and men aged 15−59 (*n* = 7790); Burkina Faso; 2003	Wealth	Prevalence	DHS household wealth index
Lakew, 2015, XS, HH	Women aged 15−49 and men aged 15−59 (*n* = 30,625); Ethiopia; 2011	Wealth	Prevalence	DHS household wealth index
Long, 2015, RXS, HH	Women and men aged 15−49 (*n* = 42,787); Tanzania; 2003−2004, 2007−2008, 2011−2012	Wealth	Prevalence	DHS household wealth index
Lucas, 2019, XS	General population (*n* = 640,654); 32 countries	Wealth	Prevalence	DHS household asset index
Mabaso, 2018, XS, HH	Adolescent girls and young women aged 15−24 (*n* = 3092); South Africa; 2012	Wealth	Prevalence	Household asset index
Magadi, 2017, XS, HH	Women aged 15−49 and men aged 15−54; Kenya; 2003, 2008	Wealth	Prevalence	DHS household wealth index
Negesse, 2021, XS, HH	Women aged 15−49 (*n* = 14,161); Ethiopia; 2016	Wealth	Prevalence	DHS household wealth index
Niragire, 2015, XS, HH	Women aged 15−49 (*n* = 6592); Rwanda; 2010	Wealth	Prevalence	DHS household wealth index
Nutor, 2020a, XS, HH	Women and men aged 15−59 (*n* = 11,270) Mozambique 2015	Wealth	Prevalence	DHS household wealth index
Nutor, 2020b, XS, HH	Women aged 15−49 and men aged 15−54 (*n* = 14,779); Malawi; 2016	Wealth	Prevalence	DHS household wealth index
Pascoe, 2015, XS, HH	Young women aged 18−22 (*n* = 2593); Zimbabwe; 2007	Wealth	Prevalence	Household asset index
Pons‐Duran, 2016, RXS, HH	Adults aged 18−50 (*n* = 1424); Mozambique; 2010, 2012	Wealth	Prevalence	Household asset index
Probst, 2017, RXS, HH	Adults aged 15 and older (*n* = 55,144); South Africa; 2005−2012	Wealth	Prevalence	Household asset index
Shah, 2022, XS, HH	Orphans and vulnerable children aged 0−17 (*n* = 1624); Haut‐Katanga and Kinshasa, Democratic Republic of the Congo; 2016−2020	Income	Prevalence	Household income <30 USD/month
Steenkamp, 2014, XS, HH	Women and men aged 18−49 (*n* = 752); Eastern Cape, South Africa; 2013	Income Wealth	Prevalence	Household asset index Household income (self‐reported last month)
Wabiri, 2013, XS, HH	Women and men aged 15 and older (*n* = 14,384); South Africa; 2008	Wealth	Prevalence	Household asset index
***Community level***				
Brodish, 2015, XS, C	Women aged 15−49 and men aged 15−59 (*n* = 43,032); Eswatini, Kenya, Lesotho, Malawi, Zambia, Zimbabwe; 2006−2011	Inequality	Prevalence	Cluster‐level Gini coefficient and wealth ratio
Feldacker, 2011, XS, C	Women aged 15−49 and men aged 15−54 (*n* = 3861); Malawi (rural); 2004	Inequality	Prevalence	Enumeration area Gini coefficient
***Multilevel***				
Durevall, 2012; XS; HH, C, SN	Women aged 15−24 (*n* = 1161); Malawi; 2004	Income Wealth Inequality	Prevalence	HH: DHS wealth index C: cluster median of wealth index; neighbourhood Gini SN: Median district consumption; District Gini
Fox, 2012; XS; HH, SN, N	General population (*n* = 138,737); Senegal, Niger, Ethiopia, Guinea, Mali, Burkina Faso, Ghana, Rwanda, Ivory Coast, Cameroon, Kenya, Tanzania, Malawi, Zimbabwe, Lesotho and Eswatini; 2002−2007	Wealth	Prevalence	HH: DHS household wealth index Regional mean wealth National Gini coefficient
Kalonda‐Kanyama, 2011; XS; HH, C	Women aged 15−49 and men aged 15−59 (*n* = 9273); Democratic Republic of the Congo; 2007	Wealth	Prevalence	DHS household wealth index Poverty incidence
Lukhele, 2016; XS; I, HH	Pregnant women aged 18 and older (*n* = 827); Eswatini; 2015	Income Wealth	Prevalence	Household asset index Individual income (self‐reported last month)
Nakazwe, 2022; RXS; HH, C	Youth aged 15−24 (2013−2014: *n* = 11,571; 2018: *n* = 10,154); Zambia, 2013−2014 and 2018	Wealth	Prevalence	DHS household wealth index Mean of wealth scores of respondents in each enumeration area

***Note***: Study designs: CC, case‐control study; CS, cohort study; ES, ecological study; RXS, repeated cross‐sectional surveys; XS, cross‐sectional. Socio‐ecological levels: C, community; HH, household; I, individual; N, national; SN, sub‐national; USD, United States Dollars.

### 3.3 Study designs

Of the included studies, the majority applied cross‐sectional (*n* = 33) or repeated cross‐sectional (*n* = 5) designs. Seven studies used a longitudinal design, including three from South Africa [34, 44, 61], two from Zimbabwe [54, 71], and one each from Namibia [33] and Uganda [70]. One study each used a case‐control [66] and an ecological [48] design.

### 3.4 Data sources and measures

Included studies utilized data from between 1985 and 2018. Most studies (*n* = 26) used Demographic and Health Survey (DHS) data. Wealth (*n* = 39) was the most common exposure measured, including at community and sub‐national levels. Fourteen studies used study‐specific measures of wealth, including constructed household wealth indices (*n* = 14) and a measure of household consumption versus the poverty line (*n* = 1). Nine studies measured income; most used self‐reported measures of household or individual income (*n* = 7), while others measured median district consumption (*n* = 1) or per capita GDP (*n* = 1). Finally, four studies measured inequality as an exposure variable; all used Gini coefficients to capture inequality at community, sub‐national or national levels.

In terms of outcomes, most studies (*n* = 42) relied on individual HIV test results. One study used UNAIDS estimates of national HIV prevalence [48], one study used self‐reported HIV status [56] and two studies relied on community‐based organization data but did not report how HIV outcomes were ascertained [44, 72]. Most studies (*n* = 41) measured prevalent HIV, while six measured incident HIV.

### 3.5 Populations

The majority (*n* = 33) of studies were focused on adolescent and adult men and women in the general population. Two studies focused on children. Six studies specifically focused on females, including two studies on adolescent girls and young women and one study each among postpartum women, pregnant women and female sex workers. Sample sizes ranged from 237 to 640,654.

### 3.6 Level of analysis

At the individual level, there were three studies with data on income and none on wealth or inequality. Forty‐two studies considered the household level, including 38 with data on wealth, three on income and one on both; no studies examined HIV and inequality at this level. Five studies considered the community level, including three with data on wealth, two on inequality and one on both; there were no studies with data on income at this level. At the sub‐national level, there was one study each with data on wealth, income and inequality. Finally, two studies included data on income and inequality at the national level, and none examined national wealth. Five studies considered two socio‐ecological levels in their assessments, and one study [38] considered three (the household, community and sub‐national levels); the remainder (*n* = 41) focused on a single level.

### 3.7 Risk of bias

Table S1 describes the ROB of the included studies. The majority (*n* = 27) had scores suggesting low ROB in all seven assessed domains (study aims and objectives, sampling, measures, statistical methods, presentation of results, interpretation of findings and ethics). Twelve of the included studies had moderate (*n* = 11) or high (*n* = 1) ROB in a single category. Five studies had moderate or high ROB in two domains, and three studies had moderate or high ROB in three domains.

Nine studies had moderate (*n* = 7) or high (*n* = 2) sampling ROB, most commonly due to the use of non‐representative sampling methods or concerns about non‐response bias. Six studies had moderate ROB in measurement, most commonly due to reliance on self‐reported data to categorize HIV status. Five studies had high ROB in their statistical approach, due to insufficient description of statistical methods, including the selection of covariates for models and the assessment of statistical significance. Three studies had high ROB in their presentation of results, primarily due to the exclusion of sample characteristics or overreliance on figures with statistical tests omitted. Finally, nine studies had moderate ROB in their interpretation of findings: of these, most omitted discussion of design or methods limitations. All included studies presented sufficient information regarding the studies’ aims and objectives and ethics.

### 3.8 HIV and wealth

Table S2 presents results from the 39 studies of wealth and HIV.

### 3.9 Household wealth and HIV incidence

Four cohort studies assessed household wealth and HIV incidence. A 2003−2005 study in South Africa [34] with moderate sampling ROB found no difference in incident HIV comparing the wealthiest to poorest terciles, but nearly twice the hazard of HIV comparing the middle to poorest tercile. A 2006−2009 study in Namibia [33] found lower odds of incident HIV in wealthier groups, but this was non‐significant in adjusted analyses. A low ROB study in Zimbabwe found no HIV incidence‐wealth relationship among women but a negative relationship among men [54]. One cohort study with low ROB conducted from 1994 to 2018 in Uganda [70] found nearly 50% lower incidence of HIV among individuals in the highest SES group compared to the lowest SES group.

Three studies assessed household wealth and HIV incidence by gender. The low ROB 1994−2018 cohort study in Uganda [70] found consistently lower HIV incidence rate among the wealthiest versus poorest quintiles for both women (40% lower) and men (52% lower). A low ROB cohort study with adults in Zimbabwe from 1998 to 2003 [54] found lower HIV incidence among wealthier men, but no difference in HIV incidence by wealth among women. And the study with adolescents and adults in Namibia [33] found lower odds of incident HIV with wealth among women in univariate analyses, but no relationship between wealth and HIV incidence among men.

### 3.10 Household wealth and HIV prevalence

Thirty‐five studies assessed household wealth and HIV prevalence. In meta‐analysis of comparable studies (Table 2 and Table S3), evidence from 15 studies (*n* = 812,238) found that individuals from the wealthiest households had 72% higher odds of living with HIV (95% confidence interval [CI]: 1.00−2.44) relative to those in the poorest households. The *Q* statistic for heterogeneity indicated variation across studies (*p*<0.001). In sex‐specific analyses, wealth was not associated with HIV prevalence.

**Table 2:** Summary of meta‐analysis results for studies of household wealth and HIV (*n* = 18)

	Pooled sample size	Pooled effect size (95% CI)	*Q*‐statistic for heterogeneity
Overall	812,238	1.72 (1.00−2.44)	836.89***
***Subgroup analyses***			
Female	101,465	1.30 (0.86−1.75)	331.30***
Male	35,979	0.98 (0.79−1.17)	3.47

*** *p*<0.001.

Overall, there was evidence of at least one positive relationship between household wealth and HIV prevalence in 20 studies, 13 with low ROB, indicating increased HIV prevalence among wealthier groups. This included a 2011 study of DHS data from over 30,000 adults in Ethiopia with low ROB [52], which found individuals in the wealthiest quintile had over four times the odds of living with HIV than those in the poorest (aOR 4.10, 95% CI 2.28−7.39). A cross‐sectional study with adults in Burkina Faso in 2003 [51] with moderate ROB found modest increases in the probability of prevalent HIV with increasing wealth (probit regression β = 0.014, *p* = 0.001). A low ROB cross‐sectional study conducted with women in Malawi in 2010 [41] found that household wealth was the strongest predictor of HIV prevalence, with women in the highest wealth quintile having nearly three times the HIV prevalence of the lowest quintile (60% vs. 22%, *p*<0.001). A pooled analysis of DHS data from 16 countries [8] with low ROB found 3% higher odds of HIV per unit increase in wealth (*p*<0.001).

Several studies with low ROB found elevated HIV prevalence comparing wealthier to middle individuals, but no difference between poorer groups. This included a 2015 study of DHS data from over 11,000 adults in Mozambique [65], which found approximately double the odds of HIV in the wealthiest versus lowest wealth quintiles, but no difference when comparing the lowest to middle or second‐lowest groups. Similar trends were observed in analyses of DHS data from Ethiopia [62], Malawi [64] and Uganda [19], and among women in Rwanda [63] and Uganda [31].

Eleven studies found at least one negative relationship between household wealth and prevalent HIV, five with low ROB, suggesting increased HIV prevalence among poorer groups. This included a repeat cross‐sectional study with over 50,000 adults in South Africa from 2005 to 2012 [69] with moderate sampling ROB, which found a higher risk of prevalent HIV among individuals with low versus high household wealth, from 36% higher in 2005 to 85% higher in 2012. A pooled analysis of DHS data from 27 countries [20] with low ROB found reduced odds of HIV prevalence in wealthier versus poorer tertiles, from 2.2% lower prevalence in middle versus poorest rural strata to 7.6% lower in richest versus poorest rural strata (*p*<0.05). Another analysis of DHS data from 32 countries [55] found small decreases in HIV prevalence with wealth (0.5% and 0.1% decreases per unit increase in wealth, *p*<0.01).

The remaining studies assessing HIV prevalence and wealth found no statistically significant differences in HIV status by household wealth, though trends were observed. Though non‐significant, longitudinal studies in Namibia [33] and South Africa [61] found higher HIV prevalence in wealthier groups, as did a cross‐sectional study in Malawi [38], while cross‐sectional studies with women in Zimbabwe [67], eSwatini [56] and South Africa [36] found lower HIV prevalence associated with wealth. A repeat cross‐sectional study with adults in Kenya [47] found a non‐significant but U‐shaped relationship between wealth and HIV.

Multiple studies presented HIV prevalence data stratified by gender, with mixed results. Only one study, an analysis of 2003−2012 DHS data from Tanzania with high ROB due to insufficient details on statistical methods and results, found a consistently positive HIV‐wealth relationship among both men and women [53]. Two others, analyses of 2003 and 2008 DHS data from Kenya with low ROB [58] and an analysis of DHS data from 32 countries with moderate ROB [55], found negative relationships between HIV and wealth among both men and women. Others found positive relationships for one gender but not the other; for example, a low ROB study of Zimbabwean households from 1998 to 2011 [71] found that HIV prevalence was associated with increased wealth among women in some years but never for men. A serial cross‐sectional study with young adults in Zambia in 2013−2014 and 2018 found null results for all groups except for young women in the middle wealth tertile in 2013−2014, for whom wealth was positively associated with HIV [60]. One multi‐country study with moderate ROB used agricultural and wage‐based measures of wealth and found that wage wealth was positively associated with HIV among both men and women in Ethiopia, but among women only in Tanzania and men only in Kenya [45]. The same study found that agricultural wealth was associated negatively with HIV for both men and women in Tanzania, for women only in Kenya and for men only in Ethiopia. The other two HIV prevalence studies with stratified analyses by gender found null results for both men and women; one had moderate ROB related to presentation of findings [50], and the other had high sampling ROB [61].

Three studies stratified their results by rural and urban settings; however, there were no clear trends in the HIV‐wealth relationship by setting. Two studies found that HIV was associated with lower wealth in both rural and urban settings [20, 58], and one study found that while HIV was not associated with wealth in urban areas, it was associated with higher wealth in rural areas [19].

### 3.11 Community wealth and HIV prevalence

A secondary analysis of 2004 DHS data from Malawi [38] with low ROB found no relationship between HIV prevalence and neighbourhood wealth. Another study with moderate ROB used 2007 DHS data to examine associations between community poverty incidence and HIV status among over 9000 adults in the Democratic Republic of the Congo [49] and found decreased prevalent HIV among individuals in poorer communities, suggesting a positive relationship with HIV and community wealth. Finally, a serial cross‐sectional study with young adults in Zambia in 2013−2014 and 2018 found decreased odds of prevalent HIV for women in wealthier communities across survey years, whereas there were increased odds of prevalent HIV for men in wealthier communities in 2013−2014 (and no relationship between HIV and community wealth for men in 2018) [60].

### 3.12 Sub‐national wealth and HIV prevalence

One low ROB study examined the relationship between sub‐national (regions within countries) mean wealth and HIV prevalence in a pooled analysis across 16 countries from 2002 to 2007 [8] and found that increased regional wealth was significantly associated with increased HIV prevalence after adjusting for regional income inequality (via Gini coefficients) and individual characteristics.

### 3.13 HIV and income

Table S4 presents results from studies of income and HIV incidence or prevalence.

### 3.14 Individual income and HIV prevalence

Three studies examined individual income and HIV prevalence. A low ROB study in Tanzania [59] observed mixed results with a moderate income (7−12 USD per month) associated with decreased HIV prevalence, while neither higher nor lower levels of income were significantly associated with HIV. Another low ROB study with women and men from a rural hospital in Nigeria [66] observed that income was inversely associated with HIV prevalence, as did a study with pregnant women in Eswatini with moderate risk of measurement bias [56, 66].

### 3.15 Household income and HIV prevalence

Four studies examined household income and HIV. One, which included postnatal women in Zimbabwe (1997−2000) and had a moderate risk of sampling and measurement bias, found that household income below 18 USD was associated with increased odds of prevalent HIV [46]. Though not statistically significant, the other two studies among the general population in South Africa, both with moderate risk of measurement bias, found reduced HIV prevalence in higher‐income households [44, 73]. Finally, a study of household wealth and HIV among orphans and vulnerable children in two parts of the Democratic Republic of the Congo found that children from households reporting a monthly income above 30 USD had increased odds of prevalent HIV [72].

### 3.16 Sub‐national income and HIV prevalence

At the sub‐national level, one cross‐sectional study with low ROB using DHS data from young women in Malawi found—though not statistically significant—that median income at the district level was associated with lower HIV prevalence [38].

### 3.17 National income and HIV incidence

At the national level, one ecological study with low ROB assessed national income and HIV incidence from 48 countries between 1985 and 2007. This study observed that higher levels of per capita GDP were associated with higher HIV incidence [48].

### 3.18 HIV and inequality

Table S5 presents results from studies of inequality and HIV prevalence. A low ROB study among young women in Malawi (2004) found that greater sub‐national inequality was associated with higher HIV prevalence [38]. One cross‐sectional study using DHS data from multiple countries (2002−2007) observed that a greater national Gini coefficient was associated with increased national HIV prevalence [8]. Another study conducted in six countries with moderate sampling ROB similarly found increased HIV prevalence associated with inequality measured through Gini coefficients [35].

### 3.19 Inequality and HIV: stratified analyses by gender

Two studies presented data stratified by gender. In an analysis of 2006−2011 DHS data from six countries with a moderate sampling ROB [35], inequality was associated with increased HIV prevalence for both men and women, although this relationship was amplified among men. In the other study, an analysis of 2004 DHS data from Malawi [42], inequality was associated with increased HIV prevalence among women only.

## 4 DISCUSSION

This is the first review to comprehensively synthesize the literature on HIV and wealth, income or inequality in SSA. We identified 47 studies from 48 countries with generally low ROB examining differences in HIV prevalence or incidence by SES at multiple socio‐ecological levels. Most studies in this area were cross‐sectional at the household level, with mixed results regarding the relationships between HIV and SES. However, economic inequality was consistently associated with increased HIV at the community, sub‐national and national levels.

### 4.1 Approaches to studying HIV and economic factors in SSA

Most studies focused on household SES, often employing the DHS household wealth index to estimate economic status in countries lacking reliable data on other indicators [75]. The use of cross‐sectional datasets such as DHS can generate useful insights about SES and HIV. However, to truly understand how economic factors affect HIV—rather than their cross‐sectional associations—additional longitudinal research is needed, particularly in light of rapidly changing HIV programme and policy contexts across SSA [70]. As access to HIV treatment is increasingly available and as policies around HIV treatment progress (e.g. expansion of Treat All approaches [76]), SES‐HIV relationships may have shifted. Studies relying only on prevalence data may, therefore, conflate increased survival among wealthier people living with HIV with increased risk among these groups [70, 77].

Most studies examined associations between SES and HIV in the general population. Findings from studies of specific populations (e.g. pregnant or postpartum women, adolescents, female sex workers, children) illustrate important differences from the general population, but these differed even within the same country; for example, one study finding decreased HIV among wealthier adolescent girls in South Africa [57], while a separate study with adolescents in Cape Town [61] found null results, as did a study with South African women [36]. These inconsistencies could be explained by differences in how (and whether) SES shapes HIV for different groups. For example, Nattrass noted household wealth explained only a small proportion of the variance in HIV prevalence among adolescents in their sample, who lived in communities where other factors may more greatly impact HIV than income or wealth. For populations disproportionately affected by HIV (e.g. youth, migrants, criminalized populations), HIV vulnerability is likely more complex than in the general population, and SES may have a more limited influence.

### 4.2 Associations between HIV and economic factors

Most studies focused on cross‐sectional relationships between HIV and wealth at the household level, but we saw no clear trends in these relationships across studies. This may reflect differences in how wealth was operationalized across settings. Meta‐analysis—which found increased odds of HIV associated with household wealth—indicated substantial heterogeneity between studies, and these results should be interpreted with caution.

About one‐third of studies found household wealth significantly associated with lower HIV prevalence, and most studies with null findings reported similar trends. Where positive correlations between wealth and HIV were present (i.e. increased HIV prevalence among wealthier groups), the strongest—or at times, only—relationships were seen when comparing the wealthiest to the poorest households. This could reflect studies being underpowered to detect differences between poorer groups, or, as others have discussed [51], these findings could reflect a threshold effect wherein greater wealth is protective against (or a risk factor for) HIV but only up to a particular level. However, inconsistent directions of associations across studies suggest that, even where such threshold effects are present, the direction of association is likely shaped by setting‐specific epidemiologic factors, such as concentration of HIV within specific social and sexual networks [78]. In some contexts, poverty may be considered both a cause and effect of HIV [79]. For instance, poverty can shape proximal (e.g. individual behaviour) as well as distal determinants to increase the risk of HIV, while HIV may drive poverty through income loss and healthcare costs related to HIV [80]. Further, in some contexts, the relationship between HIV and wealth may be non‐monotonic, with the poorest and wealthiest more affected by HIV than middle‐wealth groups [81].

Though studies on HIV and inequality were limited, inequality emerged as a stronger and more consistent correlate of HIV prevalence than individual or household income, consistent with grey literature [82]. This highlights the role of structural and area‐level deprivation, wherein individuals face limited access to high‐quality education, employment and health services while navigating heightened social and health risks. These contextual conditions, along with gendered power imbalances and segmentation of services, can elevate HIV vulnerability even among those who are not the poorest [14, 83, 84]. In the absence of consistent associations between individual or household resources and HIV outcomes, our findings suggest that addressing structural factors—particularly economic inequality—may be more impactful [85−87].

Limited subgroup analyses suggested that there may be differences in the HIV‐household wealth relationship by gender [45, 54, 71] or urbanicity [19], but the small number of studies precluded strong conclusions. Some evidence supported increased HIV prevalence in wealthier communities and sub‐national areas (e.g. country regions, districts). Scholars suggest that increased economic development in wealthier communities could foster aspirations for social mobility and demand for goods, which might increase individuals’ engagement in practices increasing exposure to HIV, such as transactional sex [14].

In terms of the association between income and HIV, evidence was more limited. At the individual level, studies consistently reported low income as a correlate of HIV [56, 59, 66]. However, two studies focused on specific vulnerable populations—pregnant women [56] and female sex workers [59]—limiting broader inferences about these relationships. These studies were further limited by reliance on self‐reported measures of income, and none included longitudinal data to examine mechanisms of effect. Individual measures of income are complicated by situations wherein individuals have differential access to and control over economic resources such that aggregate measures do not accurately convey the impacts of such resources on health [88]. This is particularly salient when considering gender norms; accurate measurement of income may be challenged by uncertainty regarding “exclusive” versus “joint” ownership of assets [89].

A notable number of the 44 included studies reported null findings (*n* = 9), including five studies of specific sub‐populations (e.g. women, adolescents) and six cross‐sectional or repeat cross‐sectional studies. This may suggest that, for certain populations, additional risk factors—beyond SES—may shape HIV risk. For example, a study of Black youth in Cape Town, South Africa [61] argued that, while SES shaped HIV risk to an extent, social influences—and the geographic concentration of HIV in neighbourhoods—may more directly shape HIV risk. It is also worth considering the role played by other dimensions of wealth not assessed, such as economic security (household or community resources to buffer economic shocks and losses) [90]. New measures of economic security, such as the Economic Security Index [90], may more appropriately reflect the fluctuating economic dynamics of households, individuals and communities. In programmatic contexts where targeting of resources to the most vulnerable households or communities is necessary, additional tools may be more effective at capturing economic risk; for example, the use of “red flag” approaches to identify specific indicators associated with vulnerability in specific contexts (e.g. not owning land or livestock) may be more appropriate than numeric scale or index scores [91].

### 4.3 Areas for future research

Our review highlights several critical areas for further research on HIV and SES. First, there is a clear need for scholarship on the influence of economic factors beyond the individual or household level. We identified a few studies examining community SES and HIV incidence or prevalence in SSA. However, some studies in SSA have found that community‐level economic factors are associated with behavioural outcomes; for example, multiple studies in South Africa have found that community poverty was associated with behaviours potentiating HIV acquisition (e.g. condomless sex, sexual concurrency) [92−94]. Further, literature from other contexts shows that cultural, social, environmental and other conditions within neighbourhoods can collectively shape HIV transmission dynamics [95, 96, 97]. Further, challenges persist regarding the conceptualization of neighbourhoods and communities, their measurement, and their effects [98, 99].

Second, there is a need to examine how SES shapes HIV prospectively. Beyond improving rigour and addressing causal inference concerns [100], this will facilitate the examination of potential mechanisms of impact and address ongoing questions around whether SES is indeed associated with HIV risk or rather with the ability to live longer with HIV, which studies of HIV prevalence cannot fully disentangle [70]. Routine surveillance approaches—including biobehavioural surveillance surveys or population‐based HIV impact assessments—can facilitate the evaluation of economic factors associated with HIV incidence through integration of recency assays or testing history methods [101]. However, these should be complemented with analyses tracking individuals over time, including through cohort studies, to establish temporality between economic factors and incident HIV.

Third, although this review focused on economic factors as potential risk factors for HIV, reverse causation—wherein HIV acquisition may lead to further changes in SES—may be a concern [79] and was not assessed here. Finally, opportunities exist to examine economic drivers of HIV among the marginalized sub‐populations in SSA. For example, despite elevated HIV prevalence among cisgender men who have sex with men and gender diverse individuals in SSA [6], we identified that no studies focused on the relationship between HIV and SES in these populations, and no studies specifically examined gender identity versus biological sex. We only identified a single study examining HIV and income among sex workers, who are disproportionately impacted by HIV in SSA [6] and for whom wealth and income are likely to shape risk (e.g. through limiting ability to negotiate condom use and necessitating engagement with multiple sex partners) [102, 103].

### 4.4 Strengths and limitations

Our review was comprehensive, including studies assessing not only HIV and wealth, but also income and economic inequality. However, our findings may have been limited by our exclusion of grey literature to allow for included articles to undergo rigorous quality checks. Grey literature may have included relevant findings related to HIV and SES, particularly inequality, which was underrepresented among peer‐reviewed papers in our review but explored in a burgeoning grey literature [82, 104]. We excluded papers examining HIV and economic factors in a single table or risk factor analysis, but not as the focus of the primary analysis; this potentially excluded relevant data, but would have substantially increased the volume of data to synthesize, given that many HIV risk factor analyses include at least one measure of SES in their sample descriptions. We limited our focus to material dimensions of SES, excluding other dimensions such as education, employment or food security. While these shape HIV outcomes, our focus was on the most direct and material pathways through which SES shapes HIV. Finally, we were limited by the quality, design and diversity of the studies included. Most studies were observational in design, precluding us from drawing causal inferences, and there was variation in the analytic methods employed and covariates included. Further, given the range of designs, outcomes and populations represented in the included studies, we were only able to perform meta‐analysis for studies of household wealth and HIV prevalence.

## 5 CONCLUSIONS

In summary, our systematic review found mixed evidence around the relationships between HIV and SES, while limited literature on economic inequality and HIV suggests that inequality is consistently associated with increased HIV prevalence and incidence. However, there are major gaps in the literature, including a need for increased longitudinal research to examine the mechanisms through which economic resources shape HIV risk, as well as an assessment of economic factors beyond the household level.

## COMPETING INTERESTS

The authors have no competing interests to declare.

## AUTHOR CONTRIBUTIONS

KA designed the study and conducted the search. KMS, HN and CEK reviewed the search strategy. KA, KMS and HN independently assessed records for eligibility and extracted the data. KA and KMS wrote the initial draft of the manuscript. All authors critically reviewed and revised the drafts and approved the final version for publication.

## FUNDING

KA was supported by the National Institute of Mental Health (F31MH124583 and R25MH083620). KMS was supported by the National Institute of Mental Health (F31MH124470 and T32MH019139). HN was supported by the National Institute of Mental Health (F31MH124535) and the National Institute on Minority Health & Health Disparities (T37MD003406).

## Supporting information



Supporting Information File 2: Risk of bias methods.
Detailed description of risk of bias assessment methods.

Supporting Information File 4: Table S2.
Comparative results from studies evaluating the relationship between HIV and wealth or poverty (*n* = 39).

Supporting Information File 3: Meta‐analysis methods and results.
Detailed description of meta‐analysis (Table S3).

Supporting Information File 5: Table S4.
Comparative results from studies evaluating the relationship between HIV and income (*n* = 8).

Supporting Information File 5: Table S5.
Comparative results from studies evaluating the relationship between HIV and inequality (*n* = 4).

Supporting Information File 1: Search strategy.
Search strings adapted for PubMed, SCOPUS, Embase, EconLit and PsycINFO databases.

## Data Availability

Data sharing is not applicable to this article as no new data were created or analysed in this study.

## References

[1] Link BG , Phelan J . Social conditions as fundamental causes of disease. J Health Soc Behav. 1995;(Spec No):80–94. 7560851

[2] Ecob R , Smith GD . Income and health: what is the nature of the relationship? Soc Sci Med. 1999;48(5):693–705. 10080369 10.1016/s0277-9536(98)00385-2

[3] Mackenbach JP , Martikainen P , Looman CW , Dalstra JA , Kunst AE , Lahelma E . The shape of the relationship between income and self‐assessed health: an international study. Int J Epidemiol. 2005;34(2):286–293. 15561750 10.1093/ije/dyh338

[4] Martikainen P , Makela P , Koskinen S , Valkonen T . Income differences in mortality: a register‐based follow‐up study of three million men and women. Int J Epidemiol. 2001;30(6):1397–1405. 11821353 10.1093/ije/30.6.1397

[5] Hargreaves JR , Bonell CP , Boler T , Boccia D , Birdthistle I , Fletcher A , et al. Systematic review exploring time trends in the association between educational attainment and risk of HIV infection in sub‐Saharan Africa. AIDS. 2008;22(3):403–414. 18195567 10.1097/QAD.0b013e3282f2aac3

[6] United Nations Joint Programme on HIV/AIDS (UNAIDS) . The path that ends AIDS: UNAIDS Global AIDS Update 2023. Geneva; 2023.

[7] World Bank . Sub‐Saharan Africa 2023. Available from: https://data.worldbank.org/region/sub‐saharan‐africa. Accessed July 15, 2024.

[8] Fox AM . The HIV‐poverty thesis re‐examined: poverty, wealth or inequality as a social determinant of HIV infection in sub‐Saharan Africa? J Biosoc Sci. 2012;44(4):459–480. 22273351 10.1017/S0021932011000745

[9] Galobardes B , Lynch J , Smith GD . Measuring socioeconomic position in health research. Br Med Bull. 2007;81–82:21–37. 10.1093/bmb/ldm001 17284541

[10] de Walque D . Does education affect HIV status? Evidence from five African countries. World Bank Econ Rev. 2009;23(2):209–233.

[11] Hargreaves JR , Glynn JR . Educational attainment and HIV‐1 infection in developing countries: a systematic review. Trop Med Int Health. 2002;7(6):489–498. 12031070 10.1046/j.1365-3156.2002.00889.x

[12] Buvé A , Bishikwabo‐Nsarhaza K , Mutangadura G . The spread and effect of HIV‐1 infection in sub‐Saharan Africa. Lancet. 2002;359(9322):2011–2017. 12076570 10.1016/S0140-6736(02)08823-2

[13] Mishra V , Assche SB , Greener R , Vaessen M , Hong R , Ghys PD , et al. HIV infection does not disproportionately affect the poorer in sub‐Saharan Africa. AIDS. 2007;21(Suppl 7):S17–28. 18040161 10.1097/01.aids.0000300532.51860.2a

[14] Fox AM . The social determinants of HIV serostatus in sub‐Saharan Africa: an inverse relationship between poverty and HIV? Public Health Rep. 2010;125(Suppl 4):16–24. 10.1177/00333549101250S405 PMC2882971 20629252

[15] Gillespie S , Kadiyala S , Greener R . Is poverty or wealth driving HIV transmission? AIDS. 2007;21(Suppl 7):S5–S16. 10.1097/01.aids.0000300531.74730.72 18040165

[16] Hajizadeh M , Sia D , Heymann SJ , Nandi A . Socioeconomic inequalities in HIV/AIDS prevalence in sub‐Saharan African countries: evidence from the Demographic Health Surveys. Int J Equity Health. 2014;13:18. 24533509 10.1186/1475-9276-13-18 PMC3930550

[17] Shelton JD , Cassell MM , Adetunji J . Is poverty or wealth at the root of HIV? Lancet. 2005;366(9491):1057–1058. 16182881 10.1016/S0140-6736(05)67401-6

[18] Rodrigo C , Rajapakse S . HIV, poverty and women. Int Health. 2010;2(1):9–16. 24037044 10.1016/j.inhe.2009.12.003

[19] Igulot P , Magadi MA . Socioeconomic status and vulnerability to HIV infection in Uganda: evidence from multilevel modelling of AIDS indicator survey data. AIDS Res Treat. 2018;2018:7812146. 29983999 10.1155/2018/7812146 PMC6011175

[20] Andrus E , Mojola SA , Moran E , Eisenberg M , Zelner J . Has the relationship between wealth and HIV risk in sub‐Saharan Africa changed over time? A temporal, gendered and hierarchical analysis. SSM—Popul Health. 2021;15:100833. 34141854 10.1016/j.ssmph.2021.100833 PMC8184650

[21] Wojcicki JM . Socioeconomic status as a risk factor for HIV infection in women in East, Central and Southern Africa: a systematic review. J Biosoc Sci. 2005;37(1):1–36. 15688569 10.1017/s0021932004006534

[22] Dinçer M , Köse N , Ünal E . The role of socioeconomic and behavioral factors in HIV‐related deaths. Hum Soc Sci Commun. 2024;11(1):1588.

[23] Woller G , Wolfe J , Brand M , Parrot L , Fowler B , Thompson J , et al. Livelihood and food security conceptual framework. Washington, DC: USAID; 2011.

[24] Swann M . Economic strengthening for HIV testing and linkage to care: a review of the evidence. AIDS Care. 2018;30(sup3):85–98. 29985055 10.1080/09540121.2018.1476665

[25] Swann M . Economic strengthening for retention in HIV care and adherence to antiretroviral therapy: a review of the evidence. AIDS Care. 2018;30(sup3):99–125. 10.1080/09540121.2018.1476665 29985055

[26] Swann M . Economic strengthening for HIV prevention and risk reduction: a review of the evidence. AIDS Care. 2018;30(sup3):37–84. 29985055 10.1080/09540121.2018.1476665

[27] Peltzer K , Pengpid S . Socioeconomic factors in adherence to HIV therapy in low‐ and middle‐income countries. J Health Popul Nutr. 2013;31(2):150–170. 23930333 10.3329/jhpn.v31i2.16379 PMC3702336

[28] Page M , McKenzie JE , Bossuyt PM , Boutron I , Hoffmann TC , Mulrow CD , et al. The PRISMA 2020 statement: an updated guideline for reporting systematic reviews. BMJ. 2021;372(71):1–9. 10.1136/bmj.n71 PMC8005924 33782057

[29] Social epidemiology. 2nd ed. New York: Oxford University Press; 2014.

[30] Downes MJ , Brennan ML , Williams HC , Dean RS . Development of a critical appraisal tool to assess the quality of cross‐sectional studies (AXIS). BMJ Open. 2016;6(12):e011458. 10.1136/bmjopen-2016-011458 PMC5168618 27932337

[31] Abimanyi‐Ochom J . The better the worse: risk factors for HIV infection among women in Kenya and Uganda: demographic and health survey. AIDS Care. 2011;23(12):1545–1550. 22117124 10.1080/09540121.2011.582477

[32] Asiedu C , Asiedu E , Owusu F . The socio‐economic determinants of HIV/AIDS infection rates in Lesotho, Malawi, Swaziland and Zimbabwe. Dev Policy Rev. 2012;30(3):305–326.

[33] Aulagnier M , Janssens W , De Beer I , van Rooy G , Gaeb E , Hesp C , et al. Incidence of HIV in Windhoek, Namibia: demographic and socio‐economic associations. PLoS One. 2011;6(10):e25860. 21991374 10.1371/journal.pone.0025860 PMC3186802

[34] Barnighausen T , Hosegood V , Timaeus IM , Newell ML . The socioeconomic determinants of HIV incidence: evidence from a longitudinal, population‐based study in rural South Africa. AIDS. 2007;21:Suppl 7(Suppl7):S29–S38. 10.1097/01.aids.0000300533.59483.95 PMC2847257 18040162

[35] Brodish PH . An association between neighbourhood wealth inequality and HIV prevalence in sub‐Saharan Africa. J Biosoc Sci. 2015;47(3):311–328. 24406021 10.1017/S0021932013000709 PMC4852138

[36] Bunyasi EW , Coetzee DJ . Relationship between socioeconomic status and HIV infection: findings from a survey in the Free State and Western Cape Provinces of South Africa. BMJ Open. 2017;7(11):e016232. 10.1136/bmjopen-2017-016232 PMC5719303 29162570

[37] Bwana VM , Simulundu E , Mboera LEG , Mfinanga SG , Michelo C . Household socio‐economic status and the risk of HIV infection among under five‐year children in Muheza district, north‐eastern Tanzania. Tanzania J Health Res. 2023;24(1):1–13.

[38] Durevall D , Lindskog A . Economic inequality and HIV in Malawi. World Dev. 2012;40(7):1435–1451.

[39] Ekholuenetale M , Onuoha H , Ekholuenetale CE , Barrow A , Nzoputam CI . Socioeconomic inequalities in human immunodeficiency virus (HIV) sero‐prevalence among women in Namibia: further analysis of population‐based data. Int J Environ Res Public Health. 2021;18(17):9397. 34501987 10.3390/ijerph18179397 PMC8431544

[40] Ekholuenetale M , Owunari Benebo F , Barrow A , Francis Idebolo A , Igwegbe Nzoputam C . Seroprevalence and determinants of human immunodeficiency virus infection among women of reproductive age in Mozambique: a multilevel analysis. Infect Dis Ther. 2020;9(4):881–900. 32910429 10.1007/s40121-020-00336-z PMC7680491

[41] Emina JBO , Madise N , Kuepie M , Zulu EM , Ye Y . Identifying HIV most‐at‐risk groups in Malawi for targeted interventions. A classification tree model. BMJ Open. 2013;3(5):e002459. 10.1136/bmjopen-2012-002459 PMC3657656 23793677

[42] Feldacker C , Ennett ST , Speizer I . It's not just who you are but where you live: an exploration of community influences on individual HIV status in rural Malawi. Soc Sci Med. 2011;72(5):717–725. 21316134 10.1016/j.socscimed.2011.01.003

[43] Fortson JG . The gradient in sub‐Saharan Africa: socioeconomic status and HIV/AIDS. Demography. 2008;45(2):303–322. 18613483 10.1353/dem.0.0006 PMC2831364

[44] Gritzman S . Is AIDS a rational disease? Some evidence from household data. South Afr J Econ. 2005;73(1):149–169.

[45] Hadley C , Maxfield A , Hruschka D . Different forms of household wealth are associated with opposing risks for HIV infection in East Africa. World Dev. 2019;113:344–351.

[46] Humphrey JH , Nathoo KJ , Hargrove JW , Iliff PJ , Mutasa KE , Moulton LH , et al. HIV‐1 and HIV‐2 prevalence and associated risk factors among postnatal women in Harare, Zimbabwe. Epidemiol Infect. 2007;135(6):933–942. 17217549 10.1017/S0950268806007709 PMC2870654

[47] Ishida K , Arnold M , Stupp P , Kizito P , Ichwara J . Exploring the connections between HIV serostatus and individual, household, and community socioeconomic resources: evidence from two population‐based surveys in Kenya. Soc Sci Med. 2012;74(2):185–195. 22169625 10.1016/j.socscimed.2011.10.019

[48] Ji T , Lin F . Income from international commodity price windfalls and HIV infections in sub‐Saharan Africa. J Afr Econ. 2017;26(5):607–624.

[49] Kalonda‐Kanyama I . Civil war, sexual violence and HIV infections: evidence from the Democratic Republic of the Congo. 2011.

[50] Kasirye I . HIV/AIDS sero‐prevalence and socio‐economic status: evidence from Uganda. Afr Dev Rev. 2016;28(3):304–318.

[51] Lachaud JP . HIV prevalence and poverty in Africa: micro‐ and macro‐econometric evidences applied to Burkina Faso. J Health Econ. 2007;26(3):483–504. 17113173 10.1016/j.jhealeco.2006.10.007

[52] Lakew Y , Benedict S , Haile D . Social determinants of HIV infection, hotspot areas and subpopulation groups in Ethiopia: evidence from the National Demographic and Health Survey in 2011. BMJ Open. 2015;5(11):e008669. 10.1136/bmjopen-2015-008669 PMC4663400 26589427

[53] Long D , Deane K . Wealthy and healthy? New evidence on the relationship between wealth and HIV vulnerability in Tanzania. Rev Afr Polit Econ. 2015;42(145):376–393.

[54] Lopman B , Lewis J , Nyamukapa C , Mushati P , Chandiwana S , Gregson S . HIV incidence and poverty in Manicaland, Zimbabwe: is HIV becoming a disease of the poor? AIDS. 2007;21(Suppl 7):S57–S66. 10.1097/01.aids.0000300536.82354.52 PMC2729486 18040166

[55] Lucas AM , Wilson NL . Schooling, wealth, risky sexual behaviour, and HIV/AIDS in sub‐Saharan Africa. J Dev Stud. 2019;55(10):2177–2192.

[56] Lukhele BW , Techasrivichien T , Suguimoto SP , Musumari PM , El‐Saaidi C , Haumba S , et al. Structural and behavioral correlates of HIV infection among pregnant women in a country with a highly generalized HIV epidemic: a cross‐sectional study with a probability sample of antenatal care facilities in Swaziland. PLoS One. 2016;11(12):e0168140. 27942014 10.1371/journal.pone.0168140 PMC5152904

[57] Mabaso M , Sokhela Z , Mohlabane N , Chibi B , Zuma K , Simbayi L . Determinants of HIV infection among adolescent girls and young women aged 15–24 years in South Africa: a 2012 population‐based national household survey. BMC Public Health. 2018;18(1):183. 29373958 10.1186/s12889-018-5051-3 PMC5787232

[58] Magadi MA . Understanding the urban‐rural disparity in HIV and poverty nexus: the case of Kenya. J Public Health. 2017;39(3):e63–e72. 10.1093/pubmed/fdw065 27412176

[59] Mizinduko MM , Moen K , Likindikoki S , Mwijage A , Leyna GH , Makyao N , et al. HIV prevalence and associated risk factors among female sex workers in Dar es Salaam, Tanzania: tracking the epidemic. Int J STD AIDS. 2020;31(10):950–957. 32772690 10.1177/0956462420917848

[60] Nakazwe C , Fylkesnes K , Michelo C , Sandøy IF . Examining the association between HIV prevalence and socioeconomic factors among young people in Zambia: do neighbourhood contextual effects play a role? PLoS One. 2022;17(6):e0268983. 35675264 10.1371/journal.pone.0268983 PMC9176771

[61] Nattrass N , Maughan‐Brown B , Seekings J , Whiteside A . Poverty, sexual behaviour, gender and HIV infection among young black men and women in Cape Town, South Africa. Afr J AIDS Res. 2012;11(4):307–317. 25860189 10.2989/16085906.2012.754830

[62] Negesse Y , Mankelkl G , Setegn M , Fetene G . Multilevel analysis of factors associated with HIV among women of reproductive age (15–49 years old) in Ethiopia: Bayesian approach. Women's Health. 2021;17:1–7. 10.1177/17455065211067638 PMC8725036 34937460

[63] Niragire F , Achia TN , Lyambabaje A , Ntaganira J . Bayesian mapping of HIV infection among women of reproductive age in Rwanda. PLoS One. 2015;10(3):e0119944. 25811462 10.1371/journal.pone.0119944 PMC4374935

[64] Nutor JJ , Duah HO , Agbadi P , Duodu PA , Gondwe KW . Spatial analysis of factors associated with HIV infection in Malawi: indicators for effective prevention. BMC Public Health. 2020;20(1):1–22. 32711500 10.1186/s12889-020-09278-0 PMC7382788

[65] Nutor JJ , Duodu PA , Agbadi P , Duah HO , Oladimeji KE , Gondwe KW . Predictors of high HIV+ prevalence in Mozambique: a complex samples logistic regression modeling and spatial mapping approaches. PLoS One. 2020;15(6):e0234034. 32497145 10.1371/journal.pone.0234034 PMC7272061

[66] Ogunmola OJ , Oladosu YO , Olamoyegun MA . Relationship between socioeconomic status and HIV infection in a rural tertiary health center. HIV AIDS (Auckl). 2014;6:61–67. 24790469 10.2147/HIV.S59061 PMC4003148

[67] Pascoe SJ , Langhaug LF , Mavhu W , Hargreaves J , Jaffar S , Hayes R , et al. Poverty, food insufficiency and HIV infection and sexual behaviour among young rural Zimbabwean women. PLoS One. 2015;10(1):e0115290. 25625868 10.1371/journal.pone.0115290 PMC4307980

[68] Pons‐Duran C , González R , Quintó L , Munguambe K , Tallada J , Naniche D , et al. Association between HIV infection and socio‐economic status: evidence from a semirural area of southern Mozambique. Trop Med Int Health. 2016;21(12):1513–1521. 27696629 10.1111/tmi.12789

[69] Probst C , Simbayi LC , Parry CDH , Shuper PA , Rehm J . Alcohol use, socioeconomic status and risk of HIV infections. AIDS Behav. 2017;21(7):1926–1937. 28352982 10.1007/s10461-017-1758-x

[70] Santelli JS , Chen I , Makumbi F , Wei Y , Nalugoda F , Lutalo T , et al. Household wealth and HIV incidence over time, rural Uganda, 1994–2018. AIDS. 2021;35(11):1835–43. 34132219 10.1097/QAD.0000000000002989 PMC8373447

[71] Schur N , Mylne A , Mushati P , Takaruza A , Ward H , Nyamukapa C , et al. The effects of household wealth on HIV prevalence in Manicaland, Zimbabwe—a prospective household census and population‐based open cohort study. J Int AIDS Soc. 2015;18:20063. 26593453 10.7448/IAS.18.1.20063 PMC4655223

[72] Shah GH , Etheredge GD , Maluantesa L , Waterfield KC , Ikhile O , Engetele E , et al. Socioeconomic status and other factors associated with HIV status among OVC in Democratic Republic of Congo (DRC). Front Public Health. 2022;10:912787. 36262234 10.3389/fpubh.2022.912787 PMC9574395

[73] Steenkamp L , Venter D , Walsh C , Dana P . Socio‐economic and demographic factors related to HIV status in urban informal settlements in the Eastern Cape, South Africa. Afr J AIDS Res. 2014;13(3):271–279. 25388981 10.2989/16085906.2014.952651

[74] Wabiri N , Taffa N . Socio‐economic inequality and HIV in South Africa. BMC Public Health. 2013;13:1037. 24180366 10.1186/1471-2458-13-1037 PMC4228412

[75] The DHS Program . Wealth Index. Available from: https://dhsprogram.com/topics/wealth‐index/. Accessed July 15, 2024.

[76] World Health Organization (WHO) . Guideline on when to start antiretroviral therapy and on pre‐exposure prophylaxis for HIV. 2015. Available from: https://www.who.int/hiv/pub/guidelines/earlyrelease‐arv/en/. Accessed August 1, 2024. 26598776

[77] Probst C , Parry CD , Rehm J . Socio‐economic differences in HIV/AIDS mortality in South Africa. Trop Med Int Health. 2016;21(7):846–855. 27118253 10.1111/tmi.12712

[78] Kenyon C , Colebunders R . Birds of a feather: homophily and sexual network structure in sub‐Saharan Africa. Int J STD AIDS. 2013;24(3):211–215. 23535354 10.1177/0956462412472455

[79] Pienaar K . Rethinking the poverty‐disease nexus: the case of HIV/AIDS in South Africa. J Med Humanit. 2017;38(3):249–266. 26687174 10.1007/s10912-015-9369-x

[80] Whiteside A . Poverty and HIV/AIDS in Africa. Third World Quart. 2002;23(2):313–332.

[81] Parkhurst JO . Understanding the correlations between wealth, poverty and human immunodeficiency virus infection in African countries. Bull World Health Organ. 2010;88(7):519–526. 20616971 10.2471/BLT.09.070185 PMC2897986

[82] Holmqvist G . HIV and income inequality: if there is a link, what does it tell us? Brasilia, DF—Brazil; 2009.

[83] Stoebenau K , Heise L , Wamoyi J , Bobrova N . Revisiting the understanding of "transactional sex" in sub‐Saharan Africa: a review and synthesis of the literature. Soc Sci Med. 2016;168:186–197. 27665064 10.1016/j.socscimed.2016.09.023

[84] Katz IT , Thomson DR , Ravishankar S , Otwombe K , Macarayan ER , Novak C , et al. Intersectional forces of urban inequality and the global HIV pandemic: a retrospective analysis. BMJ Glob Health. 2025;10(4):e014750. 10.1136/bmjgh-2023-014750 PMC11987103 40204462

[85] Gourlay A , Walker D , Singh S , Mata M , Birdthistle I . Gender‐transformative HIV and SRHR programme approaches for adolescents and young people: a realist review to inform policy and programmes. BMJ Glob Health. 2024;9(12):e014363. 10.1136/bmjgh-2023-014363 PMC11664354 39931920

[86] Toska E , Gittings L , Hodes R , Cluver LD , Govender K , Chademana KE , et al. Resourcing resilience: social protection for HIV prevention amongst children and adolescents in Eastern and Southern Africa. Afr J AIDS Res. 2016;15(2):123–140. 27399042 10.2989/16085906.2016.1194299 PMC5558245

[87] Stoner MCD , Kilburn K , Godfrey‐Faussett P , Ghys P , Pettifor AE . Cash transfers for HIV prevention: a systematic review. PLoS Med. 2021;18(11):e1003866. 34843468 10.1371/journal.pmed.1003866 PMC8668130

[88] Macintyre S , McKay L , Der G , Hiscock R . Socio‐economic position and health: what you observe depends on how you measure it. J Public Health Med. 2003;25(4):288–294. 14747587 10.1093/pubmed/fdg089

[89] Doss C , Kieran C , Kilic T . Measuring ownership, control, and use of assets. Feminist Econ. 2020;26(3):144–168.

[90] Hacker JS , Huber GA , Nichols A , Rehm P , Schlesinger M , Valletta R , et al. The economic security index: a new measure for research and policy analysis. Rev Income Wealth. 2014;60:S5–S32.

[91] Moret WM . Let's stop trying to quantify household vulnerability: the problem with simple scales for targeting and evaluating economic strengthening programs. Glob Health Sci Pract. 2018;6(1):150–160. 29496734 10.9745/GHSP-D-17-00291 PMC5878068

[92] Kalichman SC , Simbayi LC , Jooste S , Cherry C , Cain D . Poverty‐related stressors and HIV/AIDS transmission risks in two South African communities. J Urban Health. 2005;82(2):237–249. 15888636 10.1093/jurban/jti048 PMC3456564

[93] Dinkelman T , Lam D , Leibbrandt M . Household and community income, economic shocks and risky sexual behavior of young adults: evidence from the Cape Area Panel Study 2002 and 2005. AIDS. 2007;21(Suppl 7):S49–S56. 10.1097/01.aids.0000300535.05226.a9 PMC2538362 18040164

[94] Dinkelman T , Lam D , Leibbrandt M . Linking poverty and income shocks to risky sexual behaviour: evidence from a panel study of young adults in Cape Town. South Afr J Econ. 2008;76(SUPPL. 1):S52–S74. 10.1111/j.1813-6982.2008.00170.x PMC2546606 18815625

[95] Haley DF , Kramer MR , Adimora AA , Haardörfer R , Wingood GM , Ludema C , et al. Relationships between neighbourhood characteristics and current STI status among HIV‐infected and HIV‐uninfected women living in the Southern USA: a cross‐sectional multilevel analysis. Sex Transm Infect. 2017;93(8):583–589. 28270536 10.1136/sextrans-2016-052889 PMC5696110

[96] Linton SL , Cooper HLF , Luo R , Karnes C , Renneker K , Haley DF , et al. Changing places and partners: associations of neighborhood conditions with sexual network turnover among African American adults relocated from public housing. Arch Sex Behav. 2017;46(4):925–936. 26927277 10.1007/s10508-015-0687-x PMC5003751

[97] Macintyre S , Maciver S , Sooman A . Area, class and health: should we be focusing on places or people? J Soc Policy. 1993;22(2):213–234.

[98] Diez Roux AV . Neighborhoods and health: where are we and were do we go from here? Rev D'epidemiol Sante Publique. 2007;55(1):13–21. 10.1016/j.respe.2006.12.003 PMC1906739 17320330

[99] Oakes JM . The (mis)estimation of neighborhood effects: causal inference for a practicable social epidemiology. Soc Sci Med. 2004;58(10):1929–1952. 15020009 10.1016/j.socscimed.2003.08.004

[100] Rothman KJ , Greenland S . Causation and causal inference in epidemiology. Am J Public Health. 2005;95(Suppl 1):S144–S150. 16030331 10.2105/AJPH.2004.059204

[101] Gurley SA , Stupp PW , Fellows IE , Parekh BS , Young PW , Shiraishi RW , et al. Estimation of HIV‐1 incidence using a testing history‐based method; analysis from the Population‐Based HIV Impact Assessment Survey data in 12 African countries. J Acquir Immune Defic Syndr. 2023;92(3):189–196. 36730779 10.1097/QAI.0000000000003123 PMC9911103

[102] Macharia P , Moore S , Thomann M , Mwangi P , Kombo B , King R , et al. The precarity of mobile loan debt and repayment among female sex workers in Nairobi, Kenya: implications for sexual health. Glob Public Health. 2023;18(1):2184484. 36934431 10.1080/17441692.2023.2184484

[103] Nelson SH . The West's moral obligation to assist developing nations in the fight against HIV/AIDS. Health Care Anal. 2002;10(1):87–108. 15971570 10.1023/A:1015622531388

[104] Nordfors N . Economic inequality and HIV in South Africa. University of Gothenburg; 2015.

