# Supplementary material for: Wealth, income and HIV in sub‐Saharan Africa: a systematic review
Source: J Int AIDS Soc. 2025 Dec 23;28(12):e70060. doi: 10.1002/jia2.70060 (PMC12723447; doi:10.1002/jia2.70060)
Supplement: Supplementary file 1 — Supporting Information File 2: Risk of bias methods. Detailed description of risk of bias assessment methods. [file JIA2-28-e70060-s005.docx]

**Risk of Bias Methods and Results**

**Methods**

We assessed risk of bias across seven domains: (1) study aims and objectives (1 item); (2) sampling (3 items); (3) measures (2 items); (4) statistical methods (1 item); (5) presentation of results (1 item); (6) interpretation of findings (2 items); and (7) ethics (2 items). The items retained, excluded, and modified or combined are summarized in Supplemental Table 1.

For each item, we assigned a score of 0 if there were no concerns (for most items, an answer of “no” to the question) and 1 if there were concerns. We summed scores across domains and categorized studies as having low, moderate, or high risk of bias within each domain as follows:

1. Study aims and objectives (1 item): 0=low; 1=high
2. Sampling (3 items): 0=low; 1-2=moderate; 3=high
3. Measures (2 items): 0=low; 1=moderate; 2=high
4. Statistical methods (1 item): 0=low; 1=high
5. Presentation of results (1 item): 0=low; 1=high
6. Interpretation of findings (2 items): 0=low; 1=moderate; 2=high
7. Ethics (2 items): 0=low; 1=moderate; 2=high

We also generated summary scores for each study, ranging from a possible 0 to 11 points. Studies with a summary score of 0 were considered to have low risk of bias; for studies with scores ≥1, risks of bias were described in the narrative.

**Supporting Information Table 1.** Adaptations to AXIS risk of bias tool for assessment of bias in a systematic review of studies examining HIV, wealth, income, and inequality in sub-Saharan Africa.

|  | **Domain** | **AXIS Question** | **Adaptations for review** |
| --- | --- | --- | --- |
| 1 | Study Aims and Objectives | Were the aims/objectives of the study clear? | Retained |
| 2 | Study Aims and Objectives | Was the study design appropriate for the stated aim(s)? | Excluded |
| 3 | Sampling | Was the sample size justified? | Excluded |
| 4 | Sampling | Was the target/reference population clearly defined? (Is it clear who the research was about?) | Items 4-6 were combined into a single question: “Is the sample representative of the target population?” |
| 5 | Sampling | Was the sample frame taken from an appropriate population base so that it closely represented the target/reference population under investigation? |  |
| 6 | Sampling | Was the selection process likely to select subjects/participants that were representative of the target/reference population under investigation? |  |
| 7 | Sampling | Were measures undertaken to address and categorise non-responders? | Retained |
| 8 | Measures | Were the risk factor and outcome variables measured appropriate to the aims of the study? | Retained |
| 9 | Measures | Were the risk factor and outcome variables measured correctly using instruments/measurements that had been trialled, piloted or published previously? | Retained |
| 10 | Statistical Methods | Is it clear what was used to determined statistical significance and/or precision estimates? (e.g. p-values, confidence intervals) | Excluded, but considered in responses to item 11 below. |
| 11 | Statistical Methods | Were the methods (including statistical methods) sufficiently described to enable them to be repeated? | Retained |
| 12 | Presentation of Results | Were the basic data adequately described? | Items 12, 15, and 16 were combined into a single question: Were the results stated explicitly and clearly? |
| 13 | Sampling | Does the response rate raise concerns about non-response bias? | Retained |
| 14 | Sampling | If appropriate, was information about non-responders described? | Excluded |
| 15 | Presentation of Results | Were the results internally consistent? | Combined with item 12 above |
| 16 | Presentation of Results | Were the results presented for all the analyses described in the methods? | Combined with item 12 above |
| 17 | Interpretation of Findings | Were the authors' discussions and conclusions justified by the results? | Retained |
| 18 | Interpretation of Findings | Were the limitations of the study discussed? | Retained |
| 19 | Ethics | Were there any funding sources or conflicts of interest that may affect the authors’ interpretation of the results? | Retained |
| 20 | Ethics | Was ethical approval or consent of participants attained? | Retained |

**Results**

**Supplemental Table 1**. Risk of Bias of included studies (n=44).

| **Study** | **Aims^a^** | **Sampling^b^** | **Measures^c^** | **Statistical Methods^d^** | **Present-ation of results^e^** | **Interp-retation of Findings^f^** | **Ethics^g^** | Total Score |
| --- | --- | --- | --- | --- | --- | --- | --- | --- |
| **Longitudinal studies** | | | | | | | |  |
| Aulagnier, 2011 | 0 | 1 | 0 | 1 | 0 | 0 | 0 | 2 |
| Barnighausen, 2007 | 0 | 2 | 0 | 0 | 0 | 0 | 0 | 2 |
| Gritzman, 2005 | 0 | 0 | 1 | 0 | 0 | 0 | 0 | 1 |
| Lopman, 2007 | 0 | 0 | 0 | 0 | 0 | 0 | 0 | 0 |
| Nattrass, 2012 | 0 | 3 | 0 | 0 | 0 | 0 | 0 | 3 |
| Santelli, 2021 | 0 | 0 | 0 | 0 | 0 | 0 | 0 | 0 |
| Schur, 2015 | 0 | 0 | 0 | 0 | 0 | 0 | 0 | 0 |
| **Case-control studies (individual level)** | | | | | | | |  |
| Ogunmola, 2014 | 0 | 0 | 0 | 0 | 0 | 0 | 0 | 0 |
| **Ecological studies (national level)** | | | | | | | |  |
| Ji, 2017 | 0 | 0 | 0 | 0 | 0 | 0 | 0 | 0 |
| **Cross-sectional studies, by socioecological level** | | | | | | | |  |
| ***Individual level*** |  |  |  |  |  |  |  |  |
| Mizinduko, 2020 | 0 | 0 | 0 | 0 | 0 | 0 | 0 | 0 |
| ***Household level*** |  |  |  |  |  |  |  |  |
| Abimanyi-Ochom, 2011 | 0 | 0 | 0 | 0 | 0 | 0 | 0 | 0 |
| Andrus, 2021 | 0 | 0 | 0 | 0 | 0 | 0 | 0 | 0 |
| Asiedu, 2012 | 0 | 0 | 0 | 0 | 0 | 1 | 0 | 1 |
| Bunyasi, 2017 | 0 | 2 | 0 | 0 | 0 | 0 | 0 | 2 |
| Bwana, 2023 | 0 | 2 | 1 | 1 | 0 | 0 | 0 | 4 |
| Ekholuenetale, 2020 | 0 | 0 | 0 | 0 | 0 | 0 | 0 | 0 |
| Ekholuenetale, 2021 | 0 | 0 | 0 | 0 | 1 | 0 | 0 | 1 |
| Emina, 2013 | 0 | 0 | 0 | 0 | 0 | 0 | 0 | 0 |
| Fortson, 2008 | 0 | 0 | 0 | 0 | 0 | 0 | 0 | 0 |
| Hadley, 2019 | 0 | 0 | 0 | 0 | 0 | 1 | 0 | 1 |
| Humphrey, 2008 | 0 | 1 | 1 | 0 | 0 | 1 | 0 | 3 |
| Igulot, 2018 | 0 | 0 | 0 | 0 | 0 | 0 | 0 | 0 |
| Ishida, 2012 | 0 | 0 | 0 | 0 | 0 | 0 | 0 | 0 |
| Kasirye, 2016 | 0 | 0 | 0 | 0 | 0 | 1 | 0 | 1 |
| Lachaud, 2007 | 0 | 0 | 0 | 0 | 0 | 1 | 0 | 1 |
| Lakew, 2015 | 0 | 0 | 0 | 0 | 0 | 0 | 0 | 0 |
| Long, 2015 | 0 | 0 | 0 | 1 | 1 | 1 | 0 | 3 |
| Lucas, 2019 | 0 | 0 | 0 | 0 | 0 | 1 | 0 | 1 |
| Mabaso, 2018 | 0 | 0 | 0 | 0 | 0 | 0 | 0 | 0 |
| Magadi, 2017 | 0 | 0 | 0 | 0 | 0 | 0 | 0 | 0 |
| Negesse, 2021 | 0 | 0 | 0 | 0 | 0 | 0 | 0 | 0 |
| Niragire, 2015 | 0 | 0 | 0 | 0 | 0 | 0 | 0 | 0 |
| Nutor, 2020a | 0 | 0 | 0 | 0 | 0 | 0 | 0 | 0 |
| Nutor, 2020b | 0 | 0 | 0 | 0 | 0 | 0 | 0 | 0 |
| Pascoe, 2015 | 0 | 0 | 0 | 0 | 0 | 0 | 0 | 0 |
| Pons-Duran, 2016 | 0 | 0 | 0 | 0 | 0 | 0 | 0 | 0 |
| Probst, 2017 | 0 | 2 | 0 | 0 | 0 | 0 | 0 | 2 |
| Shah, 2022 | 0 | 3 | 1 | 0 | 0 | 0 | 0 | 4 |
| Steenkamp, 2014 | 0 | 0 | 1 | 1 | 0 | 0 | 0 | 2 |
| Wabiri, 2013 | 0 | 0 | 0 | 1 | 0 | 1 | 0 | 2 |
| ***Community Level*** | | | | | | | |  |
| Brodish, 2015 | 0 | 1 | 0 | 0 | 0 | 0 | 0 | 1 |
| Feldacker, 2011 | 0 | 0 | 0 | 0 | 0 | 0 | 0 | 0 |
| ***Multilevel*** | | | | | | | |  |
| Durevall, 2012 | 0 | 0 | 0 | 0 | 0 | 0 | 0 | 0 |
| Fox, 2012 | 0 | 0 | 0 | 0 | 0 | 0 | 0 | 0 |
| Kalonda-Kanyama, 2011 | 0 | 0 | 0 | 0 | 1 | 1 | 0 | 2 |
| Lukhele, 2016 | 0 | 0 | 1 | 0 | 0 | 0 | 0 | 1 |
| Nakazwe, 2022 | 0 | 0 | 0 | 0 | 0 | 0 | 0 | 0 |
| ^a^ Study aims and objectives (1 item): possible scores ranged from 0-1 where 0=low risk of bias, 1=high risk of bias.  ^b^ Sampling (3 items): possible scores ranged from 0-3 where 0=low risk of bias, 1-2=moderate risk of bias, 3=high risk of bias.  ^c^ Measures (2 items): possible scores ranged from 0-2 where 0=low risk of bias, 1=moderate risk of bias, 2=high risk of bias.  ^d^ Statistical methods (1 item): possible scores ranged from 0-1 where 0=low risk of bias, 1=high risk of bias.  ^e^ Presentation of results (1 item): possible scores ranged from 0-1 where 0=low risk of bias, 1=high risk of bias.  ^f^ Interpretation of findings (2 items): possible scores ranged from 0-2 where 0=low risk of bias, 1=moderate risk of bias, 3=high risk of bias.  ^g^ Ethics (2 items): possible scores ranged from 0-2 where 0=low risk of bias, 1=moderate risk of bias, 3=high risk of bias. | | | | | | | | |
