# Supplementary material for: Wealth, income and HIV in sub‐Saharan Africa: a systematic review
Source: J Int AIDS Soc. 2025 Dec 23;28(12):e70060. doi: 10.1002/jia2.70060 (PMC12723447; doi:10.1002/jia2.70060)
Supplement: Supplementary file 2 — Supporting Information File 4: Table S2. Comparative results from studies evaluating the relationship between HIV and wealth or poverty (n = 39). [file JIA2-28-e70060-s001.docx]

**Meta-Analysis Methods and Results**

**Methods**

*Data preparation*

We first extracted effect sizes, standard errors, sample sizes, confidence intervals from all studies using logistic or probit regression to estimate relationships between household wealth and HIV. We changed reference groups as needed to obtain consistent measures of association comparing the wealthiest and poorest wealth categories.

Where available, we used adjusted estimates. Where multiple models were applied with different adjustment sets in the same study, we used estimates from models adjusting for demographic characteristics only. Coefficients from probit regressions were transformed to log odds using the formula below and then exponentiated to obtain odds ratios for pooling.

$\log OR=\beta_{probit}\times\frac{\pi}{\surd3}$​

*Exclusion of studies from meta-analysis*

We excluded studies from meta-analysis if they reported insufficient detail for pooling of effect sizes, standard errors, or confidence intervals. We also excluded studies if two or fewer used a particular statistical analysis, independent variable, or measure.

*Missing data*

Where not reported, we contacted authors to obtain estimates for effect sizes, standard errors, or confidence intervals. If we did not receive a response, studies were excluded from meta-analysis. For multi-country studies, we contacted authors to request effect sizes for individual countries where not reported; if we did not receive a response, we included pooled effect sizes as reported in papers. Some studies using DHS data did not report sample sizes. For these studies, sample sizes were extracted from publicly available DHS reports.

*Statistical analysis*

We used random-effects models to combine odds ratios using Stata. We generated separate pooled estimates for studies measuring wealth categorically vs continuously. We also stratified pooled estimates by rurality and gender. Full model results are in Supplemental Table 3.

**Results**

*Studies included in meta-analysis*

In total, evidence from 26 studies was included in at least one meta-analysis based on comparability of independent and dependent variables. All included studies assessed household wealth and HIV prevalence using an asset-based index, and all used either logistic or probit regression. Of these, 21 measured wealth as a categorical variable (13 using DHS data) and five measured wealth as a continuous variable (Supplemental Table 2).

**Supplemental Table 2.** Summary of studies included in meta-analyses.

| **First author, publication year, study design** | **Setting (population), year of data collection** | **Independent variable measurement** | **Statistical method used** | **Included in which meta-analyses?** |
| --- | --- | --- | --- | --- |
| **Studies measuring wealth as a categorical variable** | | | | |
| ***DHS Studies*** | | | | |
| Abimanyi-Ochom, 2011, XS ^a^ | Kenya & Uganda (Women 15-49) | DHS household wealth index | Probit regression | *Categorical*: Female |
| Andrus, 2021, RXS ^b^ | 27 countries (General population) | DHS household wealth index | Logistic regression | *Categorical*: Overall (excluding Ethiopia 2011 and Uganda 2011) |
| Bunyasi, 2017, XS | South Africa (Women of reproductive age) | DHS household wealth index | Logistic regression | *Categorical*: Overall, Female |
| Durevall, 2012, XS^a^ | Malawi (Women 15-24) | DHS household wealth index | Logistic regression | *Categorical*: Female |
| Ekholuenetale, 2020, XS^a^ | Mozambique (Women 15-49) | DHS household wealth index | Logistic regression | *Categorical*: Female |
| Igulot, 2018, XS | Uganda (General population) | DHS household wealth index | Logistic regression | *Categorical*: Overall |
| Ishida, 2012, RXS^a^ | Kenya (General population) | DHS household wealth index | Logistic regression | *Categorical*: Female, Male |
| Lakew, 2015, XS | Ethiopia (General population) | DHS household wealth index | Logistic regression | *Categorical*: Overall |
| Nakazwe, 2022, RXS^a^ | Zambia (Youth 15-24) | DHS household wealth index | Logistic regression | *Categorical*: Female, Male |
| Negesse, 2021, XS | Ethiopia (Women 15-49) | DHS household wealth index | Logistic regression | *Categorical*: Overall, Female |
| Niragire, 2015, XS | Rwanda (Women 15-49) | DHS household wealth index | Logistic regression | *Categorical*: Overall, Female |
| Nutor, 2020a, XS | Mozambique (General population) | DHS household wealth index | Logistic regression | *Categorical*: Overall |
| Nutor, 2020b, XS | Malawi (General population) | DHS household wealth index | Logistic regression | *Categorical*: Overall |
| ***Non-DHS studies*** | | | | |
| Bwana, 2023, XS | Tanzania (Children under 5) | Household asset index | Logistic regression | *Categorical*: Overall |
| Lukhele, 2016; XS; HH, | Eswatini (Pregnant women) | Household asset index | Logistic regression | *Categorical*: Overall |
| Mabaso, 2018, XS | South Africa (Young women 15-24) | Household asset index | Logistic regression | *Categorical*: Overall |
| Pascoe, 2015, XS | Zimbabwe (Women 18-22) | Household asset index | Logistic regression | *Categorical*: Overall |
| Pons-Duran, 2016, RXS | Mozambique (General population) | Household asset index | Logistic regression | *Categorical*: Overall |
| Schur, 2015, CS | Zimbabwe (General population) | Household asset index | Logistic regression | *Categorical*: Overall, Female, Male |
| Wabiri, 2013, XS | South Africa (General population) | Household asset index | Logistic regression | *Categorical*: Overall |
| **Studies measuring wealth as a continuous variable** | | | | |
| Asiedu, 2012, XS | Lesotho, Malawi, Eswatini, Zimbabwe (General population) | DHS household wealth index | Probit regression coefficient | *Continuous*: Overall |
| Fox, 2012, XS | 16 countries (General population) | DHS household wealth index | Logistic regression | *Continuous*: Overall |
| Hadley, 2019, XS | Tanzania, Ethiopia, Kenya (General population) | DHS household wealth index | Logistic regression | *Continuous*: Overall, Male, Female |
| Kasirye, 2016, XS | Uganda (General population) | Household asset index | Probit regression coefficient | *Continuous*: Overall, Female, Male |
| Nattrass, 2012, CS | South Africa (Adolescents & young adults 14-22) | Household asset index | Logistic regression | *Continuous*: Overall, Female, Male |
| a Excluded from overall meta-analysis to avoid double-counting of participants represented in DHS datasets used elsewhere; only included in sex-specific analyses.  b Evidence from the 2011 DHS studies in Ethiopia and Uganda were excluded from overall meta-analysis to avoid double-counting, but included in the analyses by urbanicity. | | | | |

*Studies excluded from meta-analysis*

In total, 31 analyses from 21 studies were excluded (Supplemental Table 3). For some studies, one analysis was included while others were excluded (e.g., analyses around household wealth and HIV from the Fox 2012 article were included, while analyses around subnational wealth and national inequality were excluded based on having few or no comparable studies). The most common reasons for excluding analyses were being one of one or two studies using a particular statistical method or independent variable (22 analyses), omission of measures of effect beyond p-values (5 analyses) and omission of standard errors or confidence intervals with no response to author contact (3 analyses). One study was excluded because it relied on DHS data largely reported on in other studies.

**Supplemental Table 3.** Summary of studies excluded from meta-analysis, by outcome, socioecological level, and independent variable.

| **First author, publication year, study design, level(s) addressed, setting (population)** | **Independent variable measurement** | **Statistical method used** | **Meta-analysis exclusion reason** |
| --- | --- | --- | --- |
| **Household wealth & HIV incidence** | | | |
| Aulagnier, 2011, C, Namibia (General population) | Per capita consumption | Logistic regression | Sole study assessing per capita consumption vs poverty line |
| Barninghausen, 2007, C, South Africa (General population) | Household asset index | Cox proportional hazards | Sole study using survival analysis |
| Santelli, 2021, C, Uganda (General population) | Household asset index | Poisson regression | Only two studies assessed wealth & HIV using Poisson regression |
| **Household income & HIV incidence** | | | |
| Gritzman, 2005, C, South Africa (General population) | Per capita income | Probit regression | Sole study assessing per capita income & HIV using Poisson regression |
| **National income & HIV incidence** | | | |
| Ji, 2017, E, 48 countries (General population) | GDP per capita | Linear regression | Sole study assessing GDP per capita & HIV |
| **Individual income & HIV prevalence** | | | |
| Lukhele, 2016, XS, Swaziland (Pregnant women) | Self-reported income past month | Logistic regression | Only two studies of individual income reported odds ratios |
| Mizinduko, 2020, XS, Tanzania (Female sex workers) | Self-reported income past month | Log binomial regression | Only study of individual income reporting prevalence ratios |
| Ogunmola, 2014, CC, Nigeria (Adults in a rural hospital) | Self-reported monthly income | Logistic regression | Only two studies of individual income reported odds ratios |
| **Household wealth & HIV prevalence** | | | |
| Ekholuenetale, 2021, XS, Namibia (Women aged 15-64) | DHS household wealth index | Concentration index | Sole study using concentration index to describe disparities in HIV by wealth |
| Emina, 2013, XS, Malawi (Women aged 15-49) | DHS household wealth index | Chi-square | Analysis insufficiently robust; no measures of effect available beyond p-values |
| Fortson, 2008, XS, Burkina Faso, Cameroon, Ghana, Kenya, Tanzania (General population) | DHS household wealth index | Linear regression | One of two studies assessing wealth & HIV using linear regression |
| Kalonda-Kanyama, 2011, XS, Democratic Republic of the Congo (General population) | DHS household wealth index | Linear regression | One of two studies assessing wealth & HIV using linear regression |
| Lachaud, 2007, XS, Burkina Faso (General population) | DHS household wealth index; Gini coefficient | Probit regression | Insufficient level of detail in data (i.e., no SE or CI) + authors did not respond to contact |
| Long, 2015, RXS, Tanzania (General population) | DHS household wealth index | Chi-square | Analysis insufficiently robust; no measures of effect available beyond p-values |
| Lopman, 2007, CS, HH, Zimbabwe (General population) | Household asset index | Likelihood ratio test | Analysis insufficiently robust; no measures of effect available beyond p-values |
| Lucas, 2019, XS, 32 countries (General population) | DHS household wealth index | Logistic regression | Of 32 included DHS studies, 16 were represented in other individual studies. Pooled estimates from this study were excluded to avoid double-counting. |
| Magadi, 2017, XS, Kenya (General population) | DHS household wealth index | Logistic regression | Insufficient level of detail in data (i.e., no SE or CI) + authors did not respond to contact |
| Probst, 2017, RXS, South Africa (General population) | Household asset score | Multinomial regression | Sole study using multinomial regression |
| Steenkamp, 2014, XS, South Africa (General population) | HH asset index (grouped as poor, middle, wealthy) | Chi-square | Analysis insufficiently robust; no measures of effect reported beyond p-values |
| **Household income & HIV prevalence** | | | |
| Humphrey, 2008, XS, Zimbabwe (Postnatal women) | Family income, inflation-adjusted and converted to USD | Logistic regression | Only two studies of household income reported odds ratios |
| Shah, 2022, XS, Democratic Republic of the Congo (Orphans and vulnerable children) | Household income <30 USD/month | Logistic regression | Only two studies of household income reported odds ratios |
| Steenkamp, 2014, XS, South Africa (General population) | Self-reported monthly income | Chi-square | Analysis insufficiently robust; no measures of effect reported beyond p-values |
| **Community wealth & HIV prevalence** | | | |
| Kalonda-Kanyama, 2011, XS, Democratic Republic of the Congo (General population) | DHS household wealth index | Linear regression | Only two studies assessed community wealth |
| Nakazwe, 2022, RXS, Zambia (Young people aged 15-24) | DHS household wealth index; mean of wealth scores of respondents in the enumeration area | Logistic regression | Only two studies assessed community wealth |
| **Community inequality & HIV prevalence** | | | |
| Brodish, 2015, XS, Kenya, Lesotho, Malawi, Swaziland, Zambia, Zimbabwe (General population) | Gini coefficient and wealth ratio derived from DHS wealth index | Logistic regression | Insufficient level of detail in data for inclusion in meta-analysis (i.e., no SE or CI) + authors did not respond to contact |
| Durevall, 2012, XS, Malawi (Women aged 15-24) | cluster median of wealth index; neighborhood Gini | Logistic regression | Only two studies of community inequality provided sufficient detail for inclusion in meta-analysis. |
| Feldacker, 2011, XS, Malawi (General population) | Gini coefficient (in quartiles; categorical); percent of population below poverty line (continuous) | Logistic regression | Only two studies of community inequality provided sufficient detail for inclusion in meta-analysis. |
| **Sub-national wealth & HIV prevalence** | | | |
| Durevall, 2012, XS, Malawi (Women aged 15-24) | Median district consumption | Logistic regression | Only two studies assessed subnational wealth |
| Fox, 2012, XS, 16 countries (General population) | DHS household wealth indices | Logistic regression | Only two studies assessed subnational wealth |
| **Sub-national inequality & HIV prevalence** | | | |
| Durevall, 2012, XS, Malawi (Women aged 15-24) | District Gini | Logistic regression | Sole study of subnational inequality |
| **National inequality & HIV prevalence** | | | |
| Fox, 2012, XS, 16 countries (General population) | Gini coefficient | Logistic regression | Sole study of national inequality |

*Pooled measures of association*

Meta-analysis results are presented in Supplemental Table 4. Twenty-one studies measured wealth as a categorical variable. Of these, evidence from 15 studies representing 812,238 observations was included in the overall meta-analysis. The pooled odds ratio was 1.72 (95% CI 1.00-1.244). The Q statistic for heterogeneity indicated variation across studies (Q=836.89, p<0.001). For sex-specific analyses, we included evidence from eight studies (n=101,465) with female participants and three studies (n=35,979) with male participants. Sex-specific analyses showed no statistically significant relationship between wealth and HIV prevalence, with heterogeneity in studies with females (Q=331.30, p<0.001).

Five studies measured wealth as a continuous variable. These were included in a separate meta-analysis which found no relationship between wealth and HIV prevalence in the overall or sex-specific analyses, and heterogeneity across all studies (p<0.001).

**Supplemental Table 4.** Summary of meta-analysis results for studies of household wealth and HIV, stratified by how wealth was operationalized (categorical vs continuous).

|  | **Pooled Sample Size** | **Pooled Effect Size (95% CI)** | **Q-Statistic for Heterogeneity** |
| --- | --- | --- | --- |
| **Wealth measured categorically (n=18)** | | | |
| Overall | 812,238 | 1.72 (1.00-2.44) | 836.89*** |
| ***Stratified*** |  |  |  |
| Female | 101,465 | 1.30 (0.86-1.75) | 331.30*** |
| Male | 35,979 | 0.98 (0.79-1.17) | 3.47 |
| Rural | 430,335 | 1.38 (0.96-1.80) | 13.48 |
| Urban | 236,910 | 1.95 (1.00-2.90) | 390.98*** |
| **Wealth measured continuously (n=6)** | | | |
| Overall Pooled Effect Size | 946,492 | 1.30 (0.95-1.65) | 677.55*** |
| ***Stratified*** |  |  |  |
| Females | 504278 | 1.40 (0.92-1.89) | 67.95*** |
| Males | 253881 | 1.39 (0.61-2.17) | 213.80*** |
