# Supplementary material for: Wealth, income and HIV in sub‐Saharan Africa: a systematic review
Source: J Int AIDS Soc. 2025 Dec 23;28(12):e70060. doi: 10.1002/jia2.70060 (PMC12723447; doi:10.1002/jia2.70060)
Supplement: Supplementary file 3 — Supporting Information File 3: Meta‐analysis methods and results. Detailed description of meta‐analysis (Table S3). [file JIA2-28-e70060-s006.docx]

**Supplemental Table 3.** Comparative results from studies evaluating the relationship between HIV and wealth or poverty (n=39). *Note: Bold values indicate statistically significant findings at p<0.05. OR: odds ratio; aOR: adjusted odds ratio; HR: hazard ratio; CI: confidence interval.*

| **Study** | **Main results** | **Analyses stratified by gender or location** |
| --- | --- | --- |
| **Household Wealth and HIV Incidence (n=4)** | | |
| Aulagnier 2011 |  | **Univariate analysis, women: p=0.01 <NAD 302 (below poverty line): REF NAD 302-603 (1-2x poverty line): OR 1.1 [0.5-2.10] NAD 604-905 (2-3x poverty line): OR 0.36 [0.13-1.02] >=NAD 906 (more than 3x poverty line): OR 0.30 [0.14-0.85]**  Univariate analysis, men: p=0.82 <NAD 302 (below poverty line): REF NAD 302-603 (1-2x poverty line): OR 1.05 [0.33-3.20] NAD 604-905 (2-3x poverty line): OR 0.94 [0.24-3.64] >= NAD 906 (more than 3x poverty line): OR 0.61 [0.16-2.40]  Multivariate analysis, women: p=0.12 <NAD 302 (below poverty line): REF NAD 302-603 (1-2x poverty line): OR 0.80 [0.34-1.89] NAD 604-905 (2-3x poverty line): OR 0.52 [0.17-1.62] >=NAD 906 (more than 3x poverty line): OR 0.36 [0.15-0.89] |
| Barnighausen 2007 | Poorest 40%: REF **Middle 40%: aHR 1.7182 (0.3701), p=0.012** Wealthiest 20%: aHR 0.9249 (0.2965), p=0.808 |  |
| Lopman 2007 |  | HIV incidence among men, all ages Richest: 15.4 per 1000 person-years Poorest: 27.4 per 1000 person-years **Likelihood ratio test for trend: p=0.03**  HIV incidence among men aged 17-24 Richest: 8.3 per 1000 person-years  Poorest: 23.3 per 1000 person-years **Likelihood ratio test for trend: p=0.02**  HIV incidence among women No statistics reported, test for trend not significant |
| Santelli 2021 | Lowest SES: REF Low-middle SES: IRR 1.047, 95% CI 0.871-1.257, p=0.625 **High-middle SES: IRR 0.802, 95% CI 0.668-0.962, p=0.018 Highest SES: IRR 0.545, 95% CI 0.447-0.664, p<0.001** | Women only Lowest SES: REF Low-middle SES: IRR 1.062, 95% CI 0.829-1.362, p=0.633 High-middle SES: IRR 0.868, 95% CI 0.680-1.108, p=0.255 **Highest SES: 0.601, 95% CI 0.462-0.781, p<0.001**  Men only Lowes SES t: REF Low-middle: SES IRR 1.033, 95% CI 0.787-1.355, p=0.815 **High-middle SES: IRR 0.7233, 95% CI 0.548-0.955, p=0.022 Highest SES: IRR 0.478, 95% CI 0.353-0.647, p<0.001** |
| **Household Wealth and HIV Prevalence (n=35)** | | |
| Abimanyi-Ochom 2011 | Kenya:  Poorest: REF Poorer: β= -0.0452 (0.0319), NS Middle: β= -0.0693 (0.0229), NS Richest: β= 0.0113 (0.0529), NS  Uganda:  Poorest: REF Poorer: β= 0.0398 (0.0259), NS Middle: β= 0.0289 (0.0252), NS **Richest: β= 0.0538 (0.0273), p<0.05** |  |
| Andrus 2021 |  | **Per-year change in odds of HIV**  **Urban strata Poorest: REF Middle: aOR 0.966, p<0.05 Richest: aOR 0.929, p<0.05**  **Rural strata Poorest: REF Middle: 0.978, p<0.05 Richest: 0.924, p<0.05** |
| Asiedu 2012 | **Eswatini: β= -0.002, p=0.037**  Lesotho: β= 0.002, p=0.107  **Malawi: β= 0.006, p=0.000**  Zimbabwe: β= -0.001, p=0.381 |  |
| Bunyasi 2017 | Western Cape Province:  Poorest: REF Poorer: aOR 1.39 [0.29, 6.56] Middle: aOR 0.90 [0.08, 10.57] Richer: aOR 0.77 [0.02, 29.80] Richest: aOR 0.62 [0.00, 250.83]  Free State Province:  Poorest: REF Poorer: aOR 0.88 [0.67, 1.16] Middle: aOR 0.91 [0.72, 1.16] Richer: aOR 0.93 [0.41, 2.08] Richest: aOR 0.76 [0.16, 3.71] |  |
| Bwana 2023 | Low: REF High: OR 0.6 [0.4-1.1]  Low: REF **High: aOR 0.5 [0.3-0.9]** |  |
| Durevall 2012 ^a^ | Poorest: REF Poorer: β= -0.043 (0.405), NS Middle: β= 0.445 (0.373), NS Richer: β= 0.539 (0.378), NS Richest: β=0.259 (0.470), NS |  |
| Ekholuenetale 2020 | Poorest: REF Poorer: aOR 1.35 [0.77, 2.37] **Middle: aOR 2.02 [1.21, 3.39] Richer: aOR 3.00 [1.85, 4.87] Richest: aOR 2.64 [1.63, 4.29]** |  |
| Ekholuenetale 2021 | **Concentration index -0.258 (0.017), p<0.015** |  |
| Emina 2013 | **Household wealth was the strongest predictor of HIV prevalence among formerly married/formerly partnered women ages 30-34 and 40-49: (p<0.001).**  **HIV prevalence by wealth quintile (p<0.001): -Highest quintile: 60% -Lowest quintile: 22%** |  |
| Fortson 2008 | Model A: Linear wealth **Burkina Faso: β= 0.0369 (0.0150), p<0.05** Cameroon: β= 0.0197 (0.0147), NS **Ghana: β= -0.0153 (0.0076), p<0.05** Kenya: β= 0.0138 (0.0216), NS Tanzania: β= 0.0202 (0.0172), NS  Model B: Quadratic wealth  (β_1_: wealth; β_2_: wealth^2^) Burkina Faso: β_1_= 0.0312 (0.0349), NS; β_2_=0.0063 (0.0449), NS Cameroon: β_1_= 0.0879 (0.0494), NS;  β_2_= -0.0876 (0.0613), NS Ghana: β_1_= 0.0095 (0.0268), NS;  β_2_= -0.0304 (0.0296), NS Kenya: β_1_= 0.0320 (0.0625), NS;  β_2_= -0.0243 (0.0784), NS **Tanzania: β= 0.1637 (0.0489), p<0.05;  β_2_= -0.1839 (0.0574), p<0.05** |  |
| Fox 2012 ^a^ | **aOR 1.03 (0.014), p<0.001** |  |
| Hadley 2019 |  | Ethiopia **Women, wage: β= 1.14 [0.58, 1.71], p<0.001 Women, agri.: β= -0.35 [-0.58, -0.12], p<0.001 Men: wage: β= 1.18 [0.86, 1.50] p<0.001** Men, agri.: β= -0.30 [0.63, 0.03], NS  Kenya Women, wage: β= 0.16 [-0.16, 0.47], NS **Women, agri.: β= -0.41 [-0.65, -0.18], p<0.001 Men: wage: β= 0.46 [0.11, 0.82], p<0.001** Men, agri.: β= -0.06 [-0.35, 0.23], NS  Tanzania **Women, wage: β= 0.40 [0.23, 0.58], p<0.001 Women, agri.: β= -0.25 [-0.38, -0.12], p<0.001** Men: wage: β= 0.36 [0.04, 0.68], p<0.05 **Men, agri.: β= -0.29 [-0.48, -0.10], p<0.001** |
| Igulot 2018 | Poorest: REF Poorer: aOR 1.07 [0.92-1.23] Middle: aOR 1.06 [0.91-1.23] Richer: aOR 1.08 [0.93-1.26] **Richest: aOR 1.18 [1.00-1.38]** | Rural Poorest: REF Poorer: aOR 1.06 [0.89-1.26] Middle: aOR 1.05 [0.88-1.25] Richer: aOR 1.17 [0.98-1.39] **Richest: aOR 1.23 [1.02-1.47]**  Urban Poorest: REF Poorer: aOR 1.13 [0.87-1.46] Middle: aOR 1.18 [0.89-1.55] Richer: aOR 0.89 [0.66-1.21] Richest: aOR 0.99 [0.71-1.36] |
| Ishida 2012 |  | Women Poorest: REF Poorer: β= 0.137 [-0.181-0.455] Middle: β= 0.058 [-0.299-0.415] Richer: β= 0.097 [-0.314-0.508] Richest: β= 0.231 [-0.254-0.715]  Men Poorest: REF Poorer: β=0.098 [-0.368-0.564] Middle: β=0.017 [-0.541-0.576] Richer: β=0.212 [-0.372-0.797] Richest: β= -0.004 [-0.723-0.716] |
| Kalonda-Kanyama 2011 ^a^ | **β= 0.0356 (0.041), p<0.05** |  |
| Kasirye 2016 |  | Women: β= -0.093 (0.166), NS  Men: β= -0.178 (0.238), NS |
| Lachaud 2007 | **β= 0.014, t=2.744, p=0.001** |  |
| Lakew 2015 | Poorest: REF Poorer: aOR 0.90 [0.50-1.75] **Middle: aOR 1.70 [1.01-2.99]  Richer: aOR 2.30 [1.37-3.90] Richest: aOR 4.10 [2.28-7.39]** |  |
| Long 2015 |  | Men, 2003-2004 **Chi-square: 37.944, p<0.01** Poorest: 4.1% living with HIV Poorer: 4.3% Middle: 4.3% Richer: 7.7% Richest: 9.5%  Women, 2003-2004 **Chi-square: 91.288, p<0.01** Poorest: 2.8% Poorer: 4.6% Middle: 6.7% Richer: 11.0% Richest: 11.4%  Men, 2007-2008 **Chi-square: 18.579, p<0.01** Poorest: 3.9% Poorer: 3.7% Middle: 4.2% Richer: 4.7% Richest: 7.2%  Women, 2007-2008 **Chi-square: 28.469, p<0.01** Poorest: 5.0% Poorer: 6.6% Middle: 5.3% Richer: 6.2% Richest: 10.1%  Men, 2011-2012 **Chi-square: 11.146, p<0.01** Poorest: 3.1% Poorer: 2.9% Middle: 4.4% Richer: 3.6% Richest: 5.3%  Women, 2011-2012 **Chi-square: 33.984, p<0.01** Poorest: 4.8% Poorer: 4.8% Middle: 5.6% Richer: 7.0% Richest: 8.6% |
| Lucas 2019 |  | **Women: β= -0.005 (0.001), p<0.01**  **Men: β= -0.001 (0.001), p<0.01** |
| Lukhele 2016 | High wealth (richer & richest): REF Lower wealth (middle, poorer, & poorest): aOR 1.28 [0.88-1.84], p=0.194 |  |
| Mabaso 2018 | Quintile 1: REF **Quintile 4: aOR 0.08 [0.02-0.39], p=0.002** Quintiles 2, 3, and 5 NR |  |
| Magadi 2017 |  | Odds ratios by urbanicity:  Urban Poor: REF Middle: aOR 0.95, NS **Rich: aOR 0.62, p<0.05**  Rural Poor: REF Middle: aOR 1.28, NS **Rich: aOR 1.3 , p<0.05**  Odds ratios by gender:  Women (wealth status) Poor: REF Middle: aOR 0.88, NS **Rich: aOR 0.51, p<0.05**  Women (wealth*rurality) Rural-Poor: REF **Rural-Middle: aOR 1.60, p<0.05** **Rural-Rich: aOR 2.83, p<0.05**  Men (wealth status) Poor: REF Middle: aOR 0.73, NS **Rich: aOR 0.49, p<0.05**  Men (wealth*rurality) Rural-Poor: REF Rural-Middle: aOR 1.74, NS **Rural-Rich: aOR 3.64, p<0.05** |
| Nakazwe 2022 |  | Women, 2013-2014 Low: REF **Medium: aOR 1.61 [1.11-2.33]** High: aOR 1.26 [0.79-1.99]  Men, 2013-2014 Low: REF Medium: aOR 0.78 [0.40-1.50] High: aOR 0.51 [0.23-1.12]  Women, 2018 Low: REF Medium: aOR 1.18 [0.79-1.17] High: aOR 0.92 [0.58-1.58]  Men, 2018 Low: REF Medium: aOR 0.99 [0.42-2.37] High: aOR 1.75 [0.58-5.27] |
| Nattrass 2012 | aOR 0.95 [0.90-1.02] | Women: aOR 0.96 [0.89-1.03]  Men: aOR 0.92 [0.79-1.06] |
| Negesse 2021 | Poor (poorer & poorest): REF Middle: aOR 1.0 [0.5-2.0] **Rich (richer & richest): aOR 4.0 [3.0-6.0]** |  |
| Niragire 2015 | Low: REF Medium: posterior OR (POR) 1.135 [0.766-1.704] **High: POR 1.737 [1.064-2.835]** |  |
| Nutor 2020a | Poorest: REF Poorer: aOR 0.92 [0.68-1.25] Middle: aOR 1.21 [0.90-1.63] **Richer: aOR 2.16 [1.64-2.84] Richest: aOR 1.94 [1.47-2.56]** |  |
| Nutor 2020b | Richest: REF Rich (Middle + Richer): aOR 0.82 [0.62-1.09] **Poor (Poorest + Poorer): aOR 0.72 [0.54-0.95]** |  |
| Pascoe 2015 | Ability to afford essential items Wealthiest: REF Middle: aOR 0.92 [0.57-1.47] Poorest: aOR 0.99 [0.64-1.53] Likelihood ratio p-value: 0.926  Ownership of fixed & sellable assets Wealthiest: REF Middle: aOR 0.95 [0.58-1.55] Poorest: 1.09 [0.69-1.70] Likelihood ratio p-value: 0.839 |  |
| Pons-Duran 2016 | Poorest: REF Poorer: aOR 0.805 [0.548-1.184], p=0.270 Middle: aOR 0.892 [0.608-1.309], p=0.559 **Richer: aOR 0.595 [0.404-0.876], p=0.009 Richest: aOR 0.474 [0.316-0.711], p<0.001** |  |
| Probst 2017 | 2005 High: REF **Low: RRR 1.36 [1.10-1.69], p=0.004**  2008 High: REF **Low: RRR 1.50 [1.18-1.91], p=0.001**  2012 High: REF **Low: RRR 1.85 [1.50-2.27], p<0.001** |  |
| Schur 2015 |  | Men, 1998-2000 Lowest wealth quartile: REF Low-middle: aOR 0.91 [0.67-1.24] High-middle: aOR 1.04 [0.76-1.43] Highest: aOR 0.99 [0.67-1.49] Men, 2003-2005 Lowest: REF Low-middle: aOR 0.90 [0.66-1.23] High-middle: aOR 0.83 [0.60-1.15] Highest: aOR 0.94 [0.63-1.42] Men, 2006-2008 Lowest: REF Low-middle: aOR 1.06 [0.74-1.51] High-middle: aOR 1.01 [0.69-1.48] Highest: aOR 0.93 [0.57-1.51]  Women, 1998-2000 Lowest: REF Low-middle: aOR 0.95 [0.75-1.21] High-middle: aOR 0.82 [0.63-1.06] **Highest: aOR 0.70 [aOR 0.49-0.99]** Women, 2003-2005 Lowest: REF Low-middle: aOR 0.83 [0.688-1.00] High-middle: aOR 0.84 [0.68-1.03] Highest: aOR 0.76 [0.58-1.01] Women, 2006-2008 Lowest: REF Low-middle: aOR 0.87 [0.67-1.13] High-middle: aOR 0.87 [0.65-1.16] Highest: aOR 0.74 [0.51-1.08] |
| Steenkamp 2014 | HIV prevalence by wealth group **χ2 = 9.67, df = 2, p < 0.05** Wealthiest: 59/139 (42.4%)  Middle: 144/492 (29.3%) Poorest: 45/121 (37.2%) |  |
| Wabiri 2013 | **Richest: REF Middle: OR 3.97, p<0.01 Poorest: OR 5.48 (1.68), p<0.01** |  |
| **Community Wealth and HIV Prevalence** | | |
| Durevall 2012 ^a^ | β= 0.240 (0.203), NS |  |
| Kalonda-Kanyama 2011 ^a^ | β= -0.017 (0.019), p<0.05 |  |
| Nakazwe 2022 |  | Women, 2013-2014 Low: REF Medium: aOR 0.87 [0.55-1.37] High: aOR 0.92 [0.51-1.65]  Men, 2013-2014 Low: REF **Medium: aOR 2.53 [1.12-5.74] High: aOR 2.70 [1.03-7.08]**  Women, 2018 Low: REF **Medium: aOR 1.98 [1.17-3.35]** High: aOR 1.58 [0.72-1.56]  Men, 2018 Low: REF Medium: aOR 2.09 [0.79-5.56] High: aOR 1.97 [0.55-7.09] |
| **Sub-National Wealth and HIV Prevalence** | | |
| Fox 2012 ^a^ | **aOR 1.23 (0.238), p<0.05** |  |
| ^a^ Study presents findings at multiple socioecological levels. | | |
