# Supplementary material for: Wealth, income and HIV in sub‐Saharan Africa: a systematic review
Source: J Int AIDS Soc. 2025 Dec 23;28(12):e70060. doi: 10.1002/jia2.70060 (PMC12723447; doi:10.1002/jia2.70060)
Supplement: Supplementary file 4 — Supporting Information File 5: Table S4. Comparative results from studies evaluating the relationship between HIV and income (n = 8). [file JIA2-28-e70060-s002.docx]

**Supplemental Table 4.** Comparative results from studies evaluating the relationship between HIV and income (n=8). *Note: Bold values indicate statistically significant findings at p<0.05.*

| **Study** | **Main results** |
| --- | --- |
| **Individual income and HIV prevalence** | |
| Lukhele 2016 | At or above minimum wage: REF **Below minimum wage: aOR 1.81 [1.09-3.01], p=0.02** |
| Mizinduko 2020 | <= 10,000 TZS (US$5): REF 11,000-15,000 TZS (US$5-7): aPR 0.94 [0.55-1.60], p=0.38 **16,000-25,000 TZS (US$7-12): aPR 0.50 [0.26-0.97], p=0.04** Over 25,000 TZS (US$12+): aPR 0.78 [0.48-1.27], p=0.33 |
| Ogunmola 2014 | <40,000 NGN (US$250): REF **40,000-80,000 NGN (US$250-500): aOR 0.203 [0.070-0.588] over 80,000 NGN (US$500+): aOR 0.231 [0.072-0.746]** |
| **Household income and HIV prevalence** | |
| Humphrey 2008 | >= USD 18/month: REF **<USD 18: aOR 1.45 [1.16-1.82] Missing income data: aOR 1.18 [1.08-1.29]** |
| Gritzman 2005 | Wave 1 survey Wage income: β= -0.6564 (0.19960), NS Non-wage income: β= -0.2435 (-0.0971), NS Remittance income: β= -0.7342 (-0.2928), NS  Wave 2 survey Wage income: β= -0.-0.6916 (0.2094), NS Non-wage income: β= -0.4181 (-0.4999), NS Remittance income: β= -0.99688 (1.3729), NS |
| Shah 2022 | >= USD 30/month: REF **<USD 30: aOR 0.421 [ 0.202–0.877]** |
| Steenkamp 2014 | p-value: 0.647  No income: 9/29 (31%) living with HIV 100-500 ZAR (US$12-60): 56/153 (37%) 501-1,000 ZAR (US$60-120): 77/223 (35%) 1,001-3,000 ZAR (US$120-360): 75/239 (31%) 3,001-5,000 ZAR (US$360-600): 13/50 (26%) Over 5,000 ZAR (US$600+): 2/10 (20%) |
| **Sub-national income and HIV prevalence** | |
| Durevall 2012 | β= -0.201 (0.109), p<0.10 |
| **National income and HIV incidence** | |
| Ji 2017 | **β = 0.424 (0.142), p<0.001** |
