# Supplementary material for: Wealth, income and HIV in sub‐Saharan Africa: a systematic review
Source: J Int AIDS Soc. 2025 Dec 23;28(12):e70060. doi: 10.1002/jia2.70060 (PMC12723447; doi:10.1002/jia2.70060)
Supplement: Supplementary file 5 — Supporting Information File 5: Table S5. Comparative results from studies evaluating the relationship between HIV and inequality (n = 4). [file JIA2-28-e70060-s003.docx]

**Supplemental Table 5.** Comparative results from studies evaluating the relationship between HIV and inequality (n=4). *Note: Bold values indicate statistically significant findings at p<0.05.*

| **Study** | **Main results** | **Analyses stratified by gender or location** |
| --- | --- | --- |
| **Community inequality and HIV prevalence** | | |
| Brodish 2015 | **Gini coefficient: aOR 2.35, p<0.05** **Wealth ratio: aOR 1.32, p<0.01** | **Women only** **Gini coefficient: aOR 2.19, p<0.05** **Wealth ratio: aOR 1.25, p<0.01**  **Men only** **Gini coefficient: aOR 2.49, p<0.05** **Wealth ratio: aOR 1.36, p<0.01** |
| Durevall 2012 ^a^ | **β= 4.494 (1.591), p<0.01** |  |
| Feldacker 2011 |  | Women Gini low: REF Gini medium: aOR 1.31 [0.88-1.95], NS **Gini high: aOR 1.56 [1.02-2.42], p<0.05**  Men Gini low: REF Gini medium: aOR 1.17 [0.72-1.91], NS Gini high: 0.98 [0.54-1.79], NS |
| **Sub-national inequality and HIV prevalence** | | |
| Durevall 2012 ^a^ | **β= 6.566 (2.711), p<0.05** |  |
| **National inequality and HIV prevalence** | | |
| Fox 2012 | **aOR 2.36 (0.721), p<0.001)** |  |
| ^a^ Study presents findings at multiple socioecological levels. | | |
