# Supplementary material for: Wealth, income and HIV in sub‐Saharan Africa: a systematic review
Source: J Int AIDS Soc. 2025 Dec 23;28(12):e70060. doi: 10.1002/jia2.70060 (PMC12723447; doi:10.1002/jia2.70060)
Supplement: Supplementary file 6 — Supporting Information File 1: Search strategy. Search strings adapted for PubMed, SCOPUS, Embase, EconLit and PsycINFO databases. [file JIA2-28-e70060-s004.docx]

**Search Strings**

***PubMed:*** ("HIV"[Mesh] OR "hiv"[tw] OR "human immunodeficiency virus"[tw] OR "HIV Infections"[Mesh] OR "HIV infections"[tw] OR “HIV infection”[tw] OR "HIV-infected"[tw] OR "HIV-positive"[tw] OR "AIDS"[tw] OR "acquired immunodeficiency syndrome"[tw] OR "acquired immunologic deficiency syndrome"[tw] OR "acquired immunologic deficiency syndromes"[tw] OR "acquired immune deficiency syndrome"[tw] OR "acquired immune deficiency syndromes"[tw] OR "acquired immuno-deficiency syndrome"[tw] OR "acquired immuno deficiency syndrome"[tw] OR "acquired immuno-deficiency syndromes"[tw] OR "acquired immuno deficiency syndromes"[tw] OR "acquired immunodeficiency syndromes"[tw]) AND ("Income"[Mesh] OR "Income"[tw] OR "Gross Domestic Product"[Mesh] OR "GDP"[tw] OR "gross domestic product"[tw] OR "gross domestic products"[tw] OR "GNP"[tw] OR "gross national product"[tw] OR "gross national products"[tw] OR “gross national income”[tw] OR "Socioeconomic Factors"[Mesh:noexp] OR "Income inequality"[tw] OR "income distribution"[tw] OR "income gap"[tw] OR "economic inequality" OR "wealth inequality" OR "Poverty"[Mesh] OR "Poverty"[tw] OR "Wealth"[Mesh] OR "Wealth"[tw] OR "Economic status"[Mesh] OR "Economic status"[tw] OR "Economic development"[Mesh] OR "Economic development"[tw] OR "Economic growth"[tw]) AND (“Africa South of the Sahara”[Mesh] OR “Sub-Saharan Africa”[tw] OR “Subsaharan Africa”[tw] OR “Sub Saharan Africa”[tw] OR "Angola"[tw] OR "Angolan"[tw] OR "Benin"[tw] OR "Beninese"[tw] OR "Botswana"[tw] OR "Botswanan"[tw] OR "Burkina Faso"[tw] OR "Burkina Fasso"[tw] OR "Burkinabe"[tw] OR "Burundi"[tw] OR "Urundi"[tw] OR "Burundian"[tw] OR "Cabo Verde"[tw] OR "Cape Verde"[tw] OR "Cabo Verdean"[tw] OR "Cape Verdean"[tw] OR "Cameroon"[tw] OR "Cameroons"[tw] OR "Cameroonian"[tw] OR "Central African Republic"[tw] OR "Central African"[tw] OR "Chad"[tw] OR "Chadian"[tw] OR "Comoros"[tw] OR "Comorian"[tw] OR "Congo"[tw] OR "Congolese"[tw] OR "Cote d'Ivoire"[tw] OR "Cote dIvoire"[tw] OR "Cote diIvoire"[tw] OR "Ivory Coast"[tw] OR "Cote d'Ivoirian"[tw] OR "Democratic Republic of the Congo"[tw] OR "Zaire"[tw] OR "DRC"[tw] OR "Djibouti"[tw] OR "Djiboutian"[tw] OR "Equatorial Guinea"[tw] OR "Equatoguinean"[tw] OR "Eritrea"[tw] OR "Eritrean"[tw] OR "Ethiopia"[tw] OR "Ethiopian"[tw] OR "Gabon"[tw] OR "Gabonese Republic"[tw] OR "Gabonese"[tw] OR "Gambia"[tw] OR "Gambian"[tw] OR "Ghana"[tw] OR "Ghanaian"[tw] OR "Guinea"[tw] OR "Guinean"[tw] OR "Guinea-Bissau"[tw] OR "Guinea Bissau"[tw] OR "Guinea-Bissauan"[tw] OR "Guinea Bissauan"[tw] OR "Kenya"[tw] OR "Kenyan"[tw] OR "Lesotho"[tw] OR "Basotho"[tw] OR "Sotho"[tw] OR "Liberia"[tw] OR "Liberian"[tw] OR "Madagascar"[tw] OR "Malagasy"[tw] OR "Malawi"[tw] OR "Malawian"[tw] OR "Mali"[tw] OR "Malian"[tw] OR "Mauritania"[tw] "Mauritanian"[tw] OR "Mayotte"[tw] OR "Mozambique"[tw] OR "Mozambican"[tw] OR "Namibia"[tw] OR "Southwest Africa"[tw] OR "South West Africa"[tw] OR "Namibian"[tw] OR "Niger"[tw] OR "Nigerien"[tw] OR "Nigeria"[tw] OR "Nigerian"[tw] OR "Rwanda"[tw] OR "Ruanda"[tw] OR "Rwandan"[tw] OR "Sao Tome and Principe"[tw] OR “Sao Tome”[tw] OR "Senegal"[tw] OR "Senegalese"[tw] OR "Sierra Leone"[tw] OR "Sierra Leonean"[tw] OR "Somalia"[tw] OR "Somalian"[tw] OR "South Africa"[tw] OR "South African"[tw] OR "South Sudan"[tw] OR "South Sudanese"[tw] OR "Sudan"[tw] OR "Sudanese"[tw] OR "Swaziland"[tw] OR "eSwatini"[tw] OR "Swazi"[tw] OR "Tanzania"[tw] OR "Tanganyika"[tw] OR "Zanzibar"[tw] OR "Tanzanian"[tw] OR "Togo"[tw] OR "Togolese"[tw] OR "Uganda"[tw] OR "Ugandan"[tw] OR "Zambia"[tw] OR "Zambian"[tw] OR "Zimbabwe"[tw] OR "Zimbabwean"[tw])

***Embase:*** ('human immunodeficiency virus'/exp OR 'human immunodeficiency virus' OR 'human immunodeficiency virus infection'/exp OR 'human immunodeficiency virus infection' OR 'human immunodeficiency virus':ab,ti,kw OR 'hiv':ab,ti,kw OR 'hiv infections':ab,ti,kw OR 'hiv infection':ab,ti,kw OR 'hiv-infected':ab,ti,kw OR 'hiv-positive':ab,ti,kw OR 'acquired immune deficiency syndrome'/exp OR 'acquired immune deficiency syndrome' OR 'aids':ab,ti,kw OR 'acquired immunodeficiency syndrome':ab,ti,kw OR 'acquired immunologic deficiency syndrome':ab,ti,kw OR 'acquired immunologic deficiency syndromes':ab,ti,kw OR 'acquired immune deficiency syndrome':ab,ti,kw OR 'acquired immune deficiency syndromes':ab,ti,kw OR 'acquired immuno-deficiency syndrome':ab,ti,kw OR 'acquired immuno deficiency syndrome':ab,ti,kw OR 'acquired immuno-deficiency syndromes':ab,ti,kw OR 'acquired immuno deficiency syndromes':ab,ti,kw OR 'acquired immunodeficiency syndromes':ab,ti,kw) AND ('household income'/exp OR ‘income’:ab,ti,kw OR 'gross national income'/exp OR ‘GDP’:ab,ti,kw OR ‘gross domestic product’:ab,ti,kw OR ‘gross domestic products’:ab,ti,kw OR 'gross national product'/exp OR ‘GNP’:ab,ti,kw OR ‘gross national product’:ab,ti,kw OR ‘gross national products’:ab,ti,kw OR 'income inequality'/exp OR ‘income inequality’:ab,ti,kw OR ‘income distribution’:ab,ti,kw OR ‘income gap’:ab,ti,kw OR ‘economic inequality’ OR ‘wealth inequality’ OR 'poverty'/exp OR ‘Poverty’:ab,ti,kw OR 'wealth'/exp OR ‘wealth’:ab,ti,kw OR 'economic status'/exp OR ‘economic status’:ab,ti,kw OR 'economic development'/exp OR ‘economic development’:ab,ti,kw OR ‘economic growth’:ab,ti,kw) AND (‘Africa south of the Sahara’/exp OR ‘Sub-Saharan Africa’:ab,ti,kw OR ‘Subsaharan Africa’:ab,ti,kw OR ‘Sub Saharan Africa’:ab,ti,kw OR ‘Angola’:ab,ti,kw OR ‘Angolan’:ab,ti,kw OR ‘Benin’:ab,ti,kw OR ‘Dahomey’:ab,ti,kw OR ‘Beninese’:ab,ti,kw OR ‘Botswana’:ab,ti,kw OR ‘Botswanan’:ab,ti,kw OR ‘Burkina Faso’:ab,ti,kw OR ‘Burkina Fasso’:ab,ti,kw OR ‘Burkinabe’:ab,ti,kw OR ‘Burundi’:ab,ti,kw OR ‘Urundi’:ab,ti,kw OR ‘Burundian’:ab,ti,kw OR ‘Cabo Verde’:ab,ti,kw OR ‘Cape Verde’:ab,ti,kw OR ‘Cabo Verdean’:ab,ti,kw OR ‘Cape Verdean’:ab,ti,kw OR ‘Cameroon’:ab,ti,kw OR ‘Cameroons’:ab,ti,kw OR ‘Cameroonian’:ab,ti,kw OR ‘Central African Republic’:ab,ti,kw OR ‘Central African’:ab,ti,kw OR ‘Chad’:ab,ti,kw OR ‘Chadian’:ab,ti,kw OR ‘Comoros’:ab,ti,kw OR ‘Comorian’:ab,ti,kw OR ‘Congo’:ab,ti,kw OR ‘Congolese’:ab,ti,kw OR ‘Cote dIvoire’:ab,ti,kw OR ‘Cote diIvoire’:ab,ti,kw OR ‘Ivory Coast’:ab,ti,kw OR ‘Cote dIvoirian’:ab,ti,kw OR ‘Democratic Republic of the Congo’:ab,ti,kw OR ‘Zaire’:ab,ti,kw OR ‘DRC’:ab,ti,kw OR ‘Djibouti’:ab,ti,kw OR ‘Djiboutian’:ab,ti,kw OR ‘Equatorial Guinea’:ab,ti,kw OR ‘Equatoguinean’:ab,ti,kw OR ‘Eritrea’:ab,ti,kw OR ‘Eritrean’:ab,ti,kw OR ‘Ethiopia’:ab,ti,kw OR ‘Ethiopian’:ab,ti,kw OR ‘Gabon’:ab,ti,kw OR ‘Gabonese Republic’:ab,ti,kw OR ‘Gabonese’:ab,ti,kw OR ‘Gambia’:ab,ti,kw OR ‘Gambian’:ab,ti,kw OR ‘Ghana’:ab,ti,kw OR ‘Ghanaian’:ab,ti,kw OR ‘Guinea’:ab,ti,kw OR ‘Guinean’:ab,ti,kw OR ‘Guinea-Bissau’:ab,ti,kw OR ‘Guinea Bissau’:ab,ti,kw OR ‘Guinea-Bissauan’:ab,ti,kw OR ‘Guinea Bissauan’:ab,ti,kw OR ‘Kenya’:ab,ti,kw OR ‘Kenyan’:ab,ti,kw OR ‘Lesotho’:ab,ti,kw OR ‘Basotho’:ab,ti,kw OR ‘Sotho’:ab,ti,kw OR ‘Liberia’:ab,ti,kw OR ‘Liberian’:ab,ti,kw OR ‘Madagascar’:ab,ti,kw OR ‘Malagasy’:ab,ti,kw OR ‘Malawi’:ab,ti,kw OR ‘Malawian’:ab,ti,kw OR ‘Mali’:ab,ti,kw OR ‘Malian’:ab,ti,kw OR ‘Mauritania’:ab,ti,kw ‘Mauritanian’:ab,ti,kw OR ‘Mayotte’:ab,ti,kw OR ‘Mozambique’:ab,ti,kw OR ‘Mozambican’:ab,ti,kw OR ‘Namibia’:ab,ti,kw OR ‘Southwest Africa’:ab,ti,kw OR ‘South West Africa’:ab,ti,kw OR ‘Namibian’:ab,ti,kw OR ‘Niger’:ab,ti,kw OR ‘Nigerien’:ab,ti,kw OR ‘Nigeria’:ab,ti,kw OR ‘Nigerian’:ab,ti,kw OR ‘Rwanda’:ab,ti,kw OR ‘Ruanda’:ab,ti,kw OR ‘Rwandan’:ab,ti,kw OR ‘Sao Tome and Principe’:ab,ti,kw OR ‘Sao Tome’:ab,ti,kw OR ‘Senegal’:ab,ti,kw OR ‘Senegalese’:ab,ti,kw OR ‘Sierra Leone’:ab,ti,kw OR ‘Sierra Leonean’:ab,ti,kw OR ‘Somalia’:ab,ti,kw OR ‘Somalian’:ab,ti,kw OR ‘South Africa’:ab,ti,kw OR ‘South African’:ab,ti,kw OR ‘South Sudan’:ab,ti,kw OR ‘South Sudanese’:ab,ti,kw OR ‘Sudan’:ab,ti,kw OR ‘Sudanese’:ab,ti,kw OR ‘Swaziland’:ab,ti,kw OR ‘eSwatini’:ab,ti,kw OR ‘Swazi’:ab,ti,kw OR ‘Tanzania’:ab,ti,kw OR ‘Tanganyika’:ab,ti,kw OR ‘Zanzibar’:ab,ti,kw OR ‘Tanzanian’:ab,ti,kw OR ‘Togo’:ab,ti,kw OR ‘Togolese’:ab,ti,kw OR ‘Uganda’:ab,ti,kw OR ‘Ugandan’:ab,ti,kw OR ‘Zambia’:ab,ti,kw OR ‘Zambian’:ab,ti,kw OR ‘Zimbabwe’:ab,ti,kw OR ‘Zimbabwean’:ab,ti,kw)

***SCOPUS:*** (TITLE-ABS-KEY(“hiv”) OR TITLE-ABS-KEY(“human immunodeficiency virus”) OR TITLE-ABS-KEY(“hiv infections”) OR TITLE-ABS-KEY(“hiv infection”) OR TITLE-ABS-KEY(“hiv infected”) OR TITLE-ABS-KEY(“hiv-infected”) OR TITLE-ABS-KEY(“hiv-positive”) OR TITLE-ABS-KEY(“AIDS”) OR TITLE-ABS-KEY(“acquired immunodeficiency syndrome”) OR TITLE-ABS-KEY(“acquired immunologic deficiency syndrome”) OR TITLE-ABS-KEY(“acquired immunologic deficiency syndromes”) OR TITLE-ABS-KEY(“acquired immune deficiency syndrome”) OR TITLE-ABS-KEY(“acquired immune deficiency syndromes”) OR TITLE-ABS-KEY(“acquired immuno-deficiency syndrome”) OR TITLE-ABS-KEY(“acquired immuno deficiency syndrome”) OR TITLE-ABS-KEY(“acquired immuno-deficiency syndromes”) OR TITLE-ABS-KEY(“acquired immuno deficiency syndromes”) OR TITLE-ABS-KEY(“acquired immunodeficiency syndromes”)) AND (TITLE-ABS-KEY(“Income”) OR TITLE-ABS-KEY(“GDP”) OR TITLE-ABS-KEY(“gross domestic product”) OR TITLE-ABS-KEY(“gross domestic products”) OR TITLE-ABS-KEY(“gross national product”) OR TITLE-ABS-KEY(“gross national products”) OR TITLE-ABS-KEY(“gross national income”) OR TITLE-ABS-KEY(“Income inequality”) OR TITLE-ABS-KEY(“income distribution”) OR TITLE-ABS-KEY(“income gap”) OR TITLE-ABS-KEY(“economic inequality”) OR TITLE-ABS-KEY(“wealth inequality”) OR TITLE-ABS-KEY(“Poverty”) OR TITLE-ABS-KEY(“Wealth”) OR TITLE-ABS-KEY(“Economic status”) OR TITLE-ABS-KEY(“Economic development”) OR TITLE-ABS-KEY(“Economic growth”)) AND (TITLE-ABS-KEY(“Sub-Saharan Africa”) OR TITLE-ABS-KEY(“Subsaharan Africa”) OR TITLE-ABS-KEY(“Sub Saharan Africa”) OR TITLE-ABS-KEY(“Angola”) OR TITLE-ABS-KEY(“Angolan”) OR TITLE-ABS-KEY(“Benin”) OR TITLE-ABS-KEY(“Beninese”) OR TITLE-ABS-KEY(“Botswana”) OR TITLE-ABS-KEY(“Botswanan”) OR TITLE-ABS-KEY(“Burkina Faso”) OR TITLE-ABS-KEY(“Burkina Fasso”) OR TITLE-ABS-KEY(“Burkinabe”) OR TITLE-ABS-KEY(“Burundi”) OR TITLE-ABS-KEY(“Urundi”) OR TITLE-ABS-KEY(“Burundian”) OR TITLE-ABS-KEY(“Cabo Verde”) OR TITLE-ABS-KEY(“Cape Verde”) OR TITLE-ABS-KEY(“Cabo Verdean”) OR TITLE-ABS-KEY(“Cape Verdean”) OR TITLE-ABS-KEY(“Cameroon”) OR TITLE-ABS-KEY(“Cameroons”) OR TITLE-ABS-KEY(“Cameroonian”) OR TITLE-ABS-KEY(“Central African Republic”) OR TITLE-ABS-KEY(“Central African”) OR TITLE-ABS-KEY(“Chad”) OR TITLE-ABS-KEY(“Chadian”) OR TITLE-ABS-KEY(“Comoros”) OR TITLE-ABS-KEY(“Comorian”) OR TITLE-ABS-KEY(“Congo”) OR TITLE-ABS-KEY(“Congolese”) OR TITLE-ABS-KEY(“Cote d'Ivoire”) OR TITLE-ABS-KEY(“Cote dIvoire”) OR TITLE-ABS-KEY(“Cote diIvoire”) OR TITLE-ABS-KEY(“Ivory Coast”) OR TITLE-ABS-KEY(“Cote d'Ivoirian”) OR TITLE-ABS-KEY(“Democratic Republic of the Congo”) OR TITLE-ABS-KEY(“Zaire”) OR TITLE-ABS-KEY(“DRC”) OR TITLE-ABS-KEY(“Djibouti”) OR TITLE-ABS-KEY(“Djiboutian”) OR TITLE-ABS-KEY(“Equatorial Guinea”) OR TITLE-ABS-KEY(“Equatoguinean”) OR TITLE-ABS-KEY(“Eritrea”) OR TITLE-ABS-KEY(“Eritrean”) OR TITLE-ABS-KEY(“Ethiopia”) OR TITLE-ABS-KEY(“Ethiopian”) OR TITLE-ABS-KEY(“Gabon”) OR TITLE-ABS-KEY(“Gabonese Republic”) OR TITLE-ABS-KEY(“Gabonese”) OR TITLE-ABS-KEY(“Gambia”) OR TITLE-ABS-KEY(“Gambian”) OR TITLE-ABS-KEY(“Ghana”) OR TITLE-ABS-KEY(“Ghanaian”) OR TITLE-ABS-KEY(“Guinea”) OR TITLE-ABS-KEY(“Guinean”) OR TITLE-ABS-KEY(“Guinea-Bissau”) OR TITLE-ABS-KEY(“Guinea Bissau”) OR TITLE-ABS-KEY(“Guinea-Bissauan”) OR TITLE-ABS-KEY(“Guinea Bissauan”) OR TITLE-ABS-KEY(“Kenya”) OR TITLE-ABS-KEY(“Kenyan”) OR TITLE-ABS-KEY(“Lesotho”) OR TITLE-ABS-KEY(“Basotho”) OR TITLE-ABS-KEY(“Sotho”) OR TITLE-ABS-KEY(“Liberia”) OR TITLE-ABS-KEY(“Liberian”) OR TITLE-ABS-KEY(“Madagascar”) OR TITLE-ABS-KEY(“Malagasy”) OR TITLE-ABS-KEY(“Malawi”) OR TITLE-ABS-KEY(“Malawian”) OR TITLE-ABS-KEY(“Mali”) OR TITLE-ABS-KEY(“Malian”) OR TITLE-ABS-KEY(“Mauritania”) OR TITLE-ABS-KEY(“Mauritanian”) OR TITLE-ABS-KEY(“Mayotte”) OR TITLE-ABS-KEY(“Mozambique”) OR TITLE-ABS-KEY(“Mozambican”) OR TITLE-ABS-KEY(“Namibia”) OR TITLE-ABS-KEY(“Southwest Africa”) OR TITLE-ABS-KEY(“South West Africa”) OR TITLE-ABS-KEY(“Namibian”) OR TITLE-ABS-KEY(“Niger”) OR TITLE-ABS-KEY(“Nigerien”) OR TITLE-ABS-KEY(“Nigeria”) OR TITLE-ABS-KEY(“Nigerian”) OR TITLE-ABS-KEY(“Rwanda”) OR TITLE-ABS-KEY(“Ruanda”) OR TITLE-ABS-KEY(“Rwandan”) OR TITLE-ABS-KEY(“Sao Tome and Principe”) OR TITLE-ABS-KEY(“Sao Tome”) OR TITLE-ABS-KEY(“Senegal”) OR TITLE-ABS-KEY(“Senegalese”) OR TITLE-ABS-KEY(“Sierra Leone”) OR TITLE-ABS-KEY(“Sierra Leonean”) OR TITLE-ABS-KEY(“Somalia”) OR TITLE-ABS-KEY(“Somalian”) OR TITLE-ABS-KEY(“South Africa”) OR TITLE-ABS-KEY(“South African”) OR TITLE-ABS-KEY(“South Sudan”) OR TITLE-ABS-KEY(“South Sudanese”) OR TITLE-ABS-KEY(“Sudan”) OR TITLE-ABS-KEY(“Sudanese”) OR TITLE-ABS-KEY(“Swaziland”) OR TITLE-ABS-KEY(“eSwatini”) OR TITLE-ABS-KEY(“Swazi”) OR TITLE-ABS-KEY(“Tanzania”) OR TITLE-ABS-KEY(“Tanganyika”) OR TITLE-ABS-KEY(“Zanzibar”) OR TITLE-ABS-KEY(“Tanzanian”) OR TITLE-ABS-KEY(“Togo”) OR TITLE-ABS-KEY(“Togolese”) OR TITLE-ABS-KEY(“Uganda”) OR TITLE-ABS-KEY(“Ugandan”) OR TITLE-ABS-KEY(“Zambia”) OR TITLE-ABS-KEY(“Zambian”) OR TITLE-ABS-KEY(“Zimbabwe”) OR TITLE-ABS-KEY(“Zimbabwean”))

***PsycINFO:*** (SU “hiv” OR TI “hiv” OR AB “hiv” OR SU "human immunodeficiency virus" OR TI “human immunodeficiency virus” OR AB “human immunodeficiency virus” OR SU “HIV infections” OR TI “HIV infections” OR AB “HIV infections” OR TI “HIV infection” OR AB “HIV infection” OR SU “HIV-infected” OR TI “HIV-infected” OR AB “HIV-infected” OR SU "HIV-positive" OR TI "HIV-positive" OR AB "HIV-positive" OR SU “AIDS” OR TI “AIDS” OR AB “AIDS”OR SU "acquired immunodeficiency syndrome" OR TI "acquired immunodeficiency syndrome" OR AB "acquired immunodeficiency syndrome" OR SU "acquired immunologic deficiency syndrome" OR TI "acquired immunologic deficiency syndrome" OR AB "acquired immunologic deficiency syndrome" OR SU "acquired immunologic deficiency syndromes" OR TI "acquired immunologic deficiency syndromes" OR AB "acquired immunologic deficiency syndromes" OR SU "acquired immune deficiency syndrome" OR TI "acquired immune deficiency syndrome" OR AB "acquired immune deficiency syndrome" OR SU “acquired immune deficiency syndromes" OR TI “acquired immune deficiency syndromes" OR AB “acquired immune deficiency syndromes" OR SU "acquired immuno-deficiency syndrome" OR TI "acquired immuno-deficiency syndrome" OR AB "acquired immuno-deficiency syndrome" OR SU "acquired immuno deficiency syndrome" OR TI "acquired immuno deficiency syndrome" OR AB "acquired immuno deficiency syndrome" OR SU "acquired immuno-deficiency syndromes" OR TI "acquired immuno-deficiency syndromes" OR AB "acquired immuno-deficiency syndromes" OR SU "acquired immuno deficiency syndromes" OR TI "acquired immuno deficiency syndromes" OR AB "acquired immuno deficiency syndromes" OR SU "acquired immunodeficiency syndromes" OR TI "acquired immunodeficiency syndromes" OR AB "acquired immunodeficiency syndromes") AND (SU "income" OR TI "income" OR AB "income" OR SU "gross domestic product" OR TI "gross domestic product" OR AB "gross domestic product" OR SU "gross domestic products" OR TI "gross domestic products" OR AB "gross domestic products" OR SU "GDP" OR TI "GDP" OR AB "GDP" OR SU "gross national product" OR TI "gross national product" OR AB "gross national product" OR SU "gross national products" OR TI "gross national products" OR AB "gross national products" OR SU "GNP" OR TI "GNP" OR AB "GNP" OR SU "gross national income" OR TI "gross national income" OR AB "gross national income" OR SU "income inequality" OR TI "income inequality" OR AB "income inequality" OR SU "income distribution" OR TI "income distribution" OR AB "income distribution" OR SU "income gap" OR TI "income gap" OR AB "income gap" OR SU "economic inequality" OR TI "economic inequality" OR AB "economic inequality" OR SU "wealth inequality" OR TI "wealth inequality" OR AB "wealth inequality" OR SU "poverty" OR TI "poverty" OR AB "poverty" OR SU "wealth" OR TI "wealth" OR AB "wealth" OR SU "economic status" OR TI "economic status" OR AB "economic status" OR SU "economic development" OR TI "economic development" OR AB "economic development" OR SU "economic growth" OR TI "economic growth" OR AB "economic growth") AND (SU “Sub-Saharan Africa” OR TI “Sub-Saharan Africa” OR AB “Sub-Saharan Africa” OR SU “Subsaharan Africa” OR TI “Subsaharan Africa” OR AB “Subsaharan Africa” OR SU “Sub Saharan Africa” OR TI “Sub Saharan Africa” OR AB “Sub Saharan Africa” OR SU "Angola" OR TI "Angola" OR AB "Angola" OR SU "Angolan" OR TI "Angolan" OR AB "Angolan" OR SU "Benin" OR TI "Benin" OR AB "Benin" OR SU "Beninese" OR TI "Beninese" OR AB "Beninese" OR SU "Botswana" OR TI "Botswana" OR AB "Botswana" OR SU "Botswanan" OR TI "Botswanan" OR AB "Botswanan" OR SU "Burkina Faso" OR TI "Burkina Faso" OR AB "Burkina Faso" OR SU "Burkina Fasso" OR TI "Burkina Fasso" OR AB "Burkina Fasso" OR SU "Burkinabe" OR TI "Burkinabe" OR AB "Burkinabe" OR SU "Burundi" OR TI "Burundi" OR AB "Burundi" OR SU "Urundi" OR TI "Urundi" OR AB "Urundi" OR SU "Burundian" OR TI "Burundian" OR AB "Burundian" OR SU "Cabo Verde" OR TI "Cabo Verde" OR AB "Cabo Verde" OR SU "Cape Verde" OR TI "Cape Verde" OR AB "Cape Verde" OR SU "Cape Verdean" OR TI "Cape Verdean" OR AB "Cape Verdean" OR SU "Cameroon" OR TI "Cameroon" OR AB "Cameroon" OR SU "Cameroons" OR TI "Cameroons" OR AB "Cameroons" OR SU "Cameroonian" OR TI "Cameroonian" OR AB "Cameroonian" OR SU "Central African Republic" OR TI "Central African Republic" OR AB "Central African Republic" OR SU "Central African" OR TI "Central African" OR AB "Central African" OR SU "Chad" OR TI "Chad" OR AB "Chad" OR SU "Chadian" OR TI "Chadian" OR AB "Chadian" OR SU "Comoros" OR TI "Comoros" OR AB "Comoros" OR SU "Comorian" OR TI "Comorian" OR AB "Comorian" OR SU "Congo" OR TI "Congo" OR AB "Congo" OR SU "Congolese" OR TI "Congolese" OR AB "Congolese" OR SU "Cote d'Ivoire" OR TI "Cote d'Ivoire" OR AB "Cote d'Ivoire" OR SU Ivory Coast OR TI Ivory Coast OR AB Ivory Coast OR SU "Cote dIvoire" OR TI "Cote dIvoire" OR AB "Cote dIvoire" OR SU "Cote diIvoire" OR TI "Cote diIvoire" OR AB "Cote diIvoire" OR SU "Cote d'Ivoirian" OR TI "Cote d'Ivoirian" OR AB "Cote d'Ivoirian" OR SU "Democratic Republic of the Congo" OR TI "Democratic Republic of the Congo" OR AB "Democratic Republic of the Congo" OR SU "Zaire" OR TI "Zaire" OR AB "Zaire" OR SU "DRC" OR TI "DRC" OR AB "DRC" OR SU "Congolese" OR TI "Congolese" OR AB "Congolese" OR SU "Djibouti" OR TI "Djibouti" OR AB "Djibouti" OR SU "Djiboutian" OR TI "Djiboutian" OR AB "Djiboutian" OR SU "Equatorial Guinea" OR TI "Equatorial Guinea" OR AB "Equatorial Guinea" OR SU "Equatoguinean" OR TI "Equatoguinean" OR AB "Equatoguinean" OR SU "Eritrea" OR TI "Eritrea" OR AB "Eritrea" OR SU "Eritrean" OR TI "Eritrean" OR AB "Eritrean" OR SU "Ethiopia" OR TI "Ethiopia" OR AB "Ethiopia" OR SU "Ethiopian" OR TI "Ethiopian" OR AB "Ethiopian" OR SU "Gabon" OR TI "Gabon" OR AB "Gabon" OR SU "Gabonese Republic" OR TI "Gabonese Republic" OR AB "Gabonese Republic" OR SU "Gabonese" OR TI "Gabonese" OR AB "Gabonese" OR SU "Gambia" OR TI "Gambia" OR AB "Gambia" OR SU "Gambian" OR TI "Gambian" OR AB "Gambian" OR SU "Ghana" OR TI "Ghana" OR AB "Ghana" OR SU "Ghanaian" OR TI "Ghanaian" OR AB "Ghanaian" OR SU "Guinea" OR TI "Guinea" OR AB "Guinea" OR SU "Guinean" OR TI "Guinean" OR AB "Guinean" OR SU "Guinea-Bissau" OR TI "Guinea-Bissau" OR AB "Guinea-Bissau" OR SU "Guinea Bissau" OR TI "Guinea Bissau" OR AB "Guinea Bissau" OR SU "Guinea-Bissauan" OR TI "Guinea-Bissauan" OR AB "Guinea-Bissauan" OR SU "Kenya" OR TI "Kenya" OR AB "Kenya" OR SU "Kenyan" OR TI "Kenyan" OR AB "Kenyan" OR SU "Lesotho" OR TI "Lesotho" OR AB "Lesotho" OR SU "Basotho" OR TI "Basotho" OR AB "Basotho" OR SU “Sotho” OR TI “Sotho” OR AB “Sotho” OR SU "Liberia" OR TI "Liberia" OR AB "Liberia" OR SU "Liberian" OR TI "Liberian" OR AB "Liberian" OR SU "Madagascar" OR TI "Madagascar" OR AB "Madagascar" OR SU "Malagasy" OR TI "Malagasy" OR AB "Malagasy" OR SU "Malawi" OR TI "Malawi" OR AB "Malawi" OR SU "Malawian" OR TI "Malawian" OR AB "Malawian" OR SU "Mali" OR TI "Mali" OR AB "Mali" OR SU "Malian" OR TI "Malian" OR AB "Malian" OR SU "Mauritania" OR TI "Mauritania" OR AB "Mauritania" OR SU "Mauritanian" OR TI "Mauritanian" OR AB "Mauritanian" OR SU "Mayotte" OR TI "Mayotte" OR AB "Mayotte" OR SU "Mozambique" OR TI "Mozambique" OR AB "Mozambique" OR SU "Mozambican" OR TI "Mozambican" OR AB "Mozambican" OR SU "Namibia" OR TI "Namibia" OR AB "Namibia" OR SU "Southwest Africa" OR TI "Southwest Africa" OR AB "Southwest Africa" OR SU "South West Africa" OR TI "South West Africa" OR AB "South West Africa" OR SU "Namibian" OR TI "Namibian" OR AB "Namibian" OR SU "Niger" OR TI "Niger" OR AB "Niger" OR SU "Nigerien" OR TI "Nigerien" OR AB "Nigerien" OR SU "Nigeria" OR TI "Nigeria" OR AB "Nigeria" OR SU "Nigerian" OR TI "Nigerian" OR AB "Nigerian" OR SU "Rwanda" OR TI "Rwanda" OR AB "Rwanda" OR SU "Ruanda" OR TI "Ruanda" OR AB "Ruanda" OR SU "Rwandan" OR TI "Rwandan" OR AB "Rwandan" OR SU "Sao Tome and Principe" OR TI "Sao Tome and Principe" OR AB "Sao Tome and Principe" OR SU "Senegal" OR TI "Senegal" OR AB "Senegal" OR SU "Senegalese" OR TI "Senegalese" OR AB "Senegalese" OR SU "Sierra Leone" OR TI "Sierra Leone" OR AB "Sierra Leone" OR SU "Sierra Leonean" OR TI "Sierra Leonean" OR AB "Sierra Leonean" OR SU "Somalia" OR TI "Somalia" OR AB "Somalia" OR SU "Somalian" OR TI "Somalian" OR AB "Somalian" OR SU "South Africa" OR TI "South Africa" OR AB "South Africa" OR SU "South African" OR TI "South African" OR AB "South African" OR SU "South Sudan" OR TI "South Sudan" OR AB "South Sudan" OR SU "South Sudanese" OR TI "South Sudanese" OR AB "South Sudanese" OR SU "Sudan" OR TI "Sudan" OR AB "Sudan" OR SU "Sudanese" OR TI "Sudanese" OR AB "Sudanese" OR SU "Swaziland" OR TI "Swaziland" OR AB "Swaziland" OR SU "eSwatini" OR TI "eSwatini" OR AB "eSwatini" OR SU "Swazi" OR TI "Swazi" OR AB "Swazi" OR SU "Tanzania" OR TI "Tanzania" OR AB "Tanzania" OR SU "Tanganyika" OR TI "Tanganyika" OR AB "Tanganyika" OR SU "Zanzibar" OR TI "Zanzibar" OR AB "Zanzibar" OR SU "Tanzanian" OR TI "Tanzanian" OR AB "Tanzanian" OR SU "Togo" OR TI "Togo" OR AB "Togo" OR SU "Togolese" OR TI "Togolese" OR AB "Togolese" OR SU "Uganda" OR TI "Uganda" OR AB "Uganda" OR SU "Ugandan" OR TI "Ugandan" OR AB "Ugandan" OR SU "Zambia" OR TI "Zambia" OR AB "Zambia" OR SU "Zambian" OR TI "Zambian" OR AB "Zambian" OR SU "Zimbabwe" OR TI "Zimbabwe" OR AB "Zimbabwe" OR SU "Zimbabwean" OR TI "Zimbabwean" OR AB "Zimbabwean”)

***EconLit:*** (SU “hiv” OR TI “hiv” OR AB “hiv” OR SU "human immunodeficiency virus" OR TI “human immunodeficiency virus” OR AB “human immunodeficiency virus” OR SU “HIV infections” OR TI “HIV infections” OR AB “HIV infections” OR TI “HIV infection” OR AB “HIV infection” OR SU “HIV-infected” OR TI “HIV-infected” OR AB “HIV-infected” OR SU "HIV-positive" OR TI "HIV-positive" OR AB "HIV-positive" OR SU “AIDS” OR TI “AIDS” OR AB “AIDS”OR SU "acquired immunodeficiency syndrome" OR TI "acquired immunodeficiency syndrome" OR AB "acquired immunodeficiency syndrome" OR SU "acquired immunologic deficiency syndrome" OR TI "acquired immunologic deficiency syndrome" OR AB "acquired immunologic deficiency syndrome" OR SU "acquired immunologic deficiency syndromes" OR TI "acquired immunologic deficiency syndromes" OR AB "acquired immunologic deficiency syndromes" OR SU "acquired immune deficiency syndrome" OR TI "acquired immune deficiency syndrome" OR AB "acquired immune deficiency syndrome" OR SU “acquired immune deficiency syndromes" OR TI “acquired immune deficiency syndromes" OR AB “acquired immune deficiency syndromes" OR SU "acquired immuno-deficiency syndrome" OR TI "acquired immuno-deficiency syndrome" OR AB "acquired immuno-deficiency syndrome" OR SU "acquired immuno deficiency syndrome" OR TI "acquired immuno deficiency syndrome" OR AB "acquired immuno deficiency syndrome" OR SU "acquired immuno-deficiency syndromes" OR TI "acquired immuno-deficiency syndromes" OR AB "acquired immuno-deficiency syndromes" OR SU "acquired immuno deficiency syndromes" OR TI "acquired immuno deficiency syndromes" OR AB "acquired immuno deficiency syndromes" OR SU "acquired immunodeficiency syndromes" OR TI "acquired immunodeficiency syndromes" OR AB "acquired immunodeficiency syndromes") AND (SU "income" OR TI "income" OR AB "income" OR SU "gross domestic product" OR TI "gross domestic product" OR AB "gross domestic product" OR SU "gross domestic products" OR TI "gross domestic products" OR AB "gross domestic products" OR SU "GDP" OR TI "GDP" OR AB "GDP" OR SU "gross national product" OR TI "gross national product" OR AB "gross national product" OR SU "gross national products" OR TI "gross national products" OR AB "gross national products" OR SU "GNP" OR TI "GNP" OR AB "GNP" OR SU "gross national income" OR TI "gross national income" OR AB "gross national income" OR SU "income inequality" OR TI "income inequality" OR AB "income inequality" OR SU "income distribution" OR TI "income distribution" OR AB "income distribution" OR SU "income gap" OR TI "income gap" OR AB "income gap" OR SU "economic inequality" OR TI "economic inequality" OR AB "economic inequality" OR SU "wealth inequality" OR TI "wealth inequality" OR AB "wealth inequality" OR SU "poverty" OR TI "poverty" OR AB "poverty" OR SU "wealth" OR TI "wealth" OR AB "wealth" OR SU "economic status" OR TI "economic status" OR AB "economic status" OR SU "economic development" OR TI "economic development" OR AB "economic development" OR SU "economic growth" OR TI "economic growth" OR AB "economic growth") AND (SU “Sub-Saharan Africa” OR TI “Sub-Saharan Africa” OR AB “Sub-Saharan Africa” OR SU “Subsaharan Africa” OR TI “Subsaharan Africa” OR AB “Subsaharan Africa” OR SU “Sub Saharan Africa” OR TI “Sub Saharan Africa” OR AB “Sub Saharan Africa” OR SU "Angola" OR TI "Angola" OR AB "Angola" OR SU "Angolan" OR TI "Angolan" OR AB "Angolan" OR SU "Benin" OR TI "Benin" OR AB "Benin" OR SU "Beninese" OR TI "Beninese" OR AB "Beninese" OR SU "Botswana" OR TI "Botswana" OR AB "Botswana" OR SU "Botswanan" OR TI "Botswanan" OR AB "Botswanan" OR SU "Burkina Faso" OR TI "Burkina Faso" OR AB "Burkina Faso" OR SU "Burkina Fasso" OR TI "Burkina Fasso" OR AB "Burkina Fasso" OR SU "Burkinabe" OR TI "Burkinabe" OR AB "Burkinabe" OR SU "Burundi" OR TI "Burundi" OR AB "Burundi" OR SU "Urundi" OR TI "Urundi" OR AB "Urundi" OR SU "Burundian" OR TI "Burundian" OR AB "Burundian" OR SU "Cabo Verde" OR TI "Cabo Verde" OR AB "Cabo Verde" OR SU "Cape Verde" OR TI "Cape Verde" OR AB "Cape Verde" OR SU "Cape Verdean" OR TI "Cape Verdean" OR AB "Cape Verdean" OR SU "Cameroon" OR TI "Cameroon" OR AB "Cameroon" OR SU "Cameroons" OR TI "Cameroons" OR AB "Cameroons" OR SU "Cameroonian" OR TI "Cameroonian" OR AB "Cameroonian" OR SU "Central African Republic" OR TI "Central African Republic" OR AB "Central African Republic" OR SU "Central African" OR TI "Central African" OR AB "Central African" OR SU "Chad" OR TI "Chad" OR AB "Chad" OR SU "Chadian" OR TI "Chadian" OR AB "Chadian" OR SU "Comoros" OR TI "Comoros" OR AB "Comoros" OR SU "Comorian" OR TI "Comorian" OR AB "Comorian" OR SU "Congo" OR TI "Congo" OR AB "Congo" OR SU "Congolese" OR TI "Congolese" OR AB "Congolese" OR SU "Cote d'Ivoire" OR TI "Cote d'Ivoire" OR AB "Cote d'Ivoire" OR SU Ivory Coast OR TI Ivory Coast OR AB Ivory Coast OR SU "Cote dIvoire" OR TI "Cote dIvoire" OR AB "Cote dIvoire" OR SU "Cote diIvoire" OR TI "Cote diIvoire" OR AB "Cote diIvoire" OR SU "Cote d'Ivoirian" OR TI "Cote d'Ivoirian" OR AB "Cote d'Ivoirian" OR SU "Democratic Republic of the Congo" OR TI "Democratic Republic of the Congo" OR AB "Democratic Republic of the Congo" OR SU "Zaire" OR TI "Zaire" OR AB "Zaire" OR SU "DRC" OR TI "DRC" OR AB "DRC" OR SU "Congolese" OR TI "Congolese" OR AB "Congolese" OR SU "Djibouti" OR TI "Djibouti" OR AB "Djibouti" OR SU "Djiboutian" OR TI "Djiboutian" OR AB "Djiboutian" OR SU "Equatorial Guinea" OR TI "Equatorial Guinea" OR AB "Equatorial Guinea" OR SU "Equatoguinean" OR TI "Equatoguinean" OR AB "Equatoguinean" OR SU "Eritrea" OR TI "Eritrea" OR AB "Eritrea" OR SU "Eritrean" OR TI "Eritrean" OR AB "Eritrean" OR SU "Ethiopia" OR TI "Ethiopia" OR AB "Ethiopia" OR SU "Ethiopian" OR TI "Ethiopian" OR AB "Ethiopian" OR SU "Gabon" OR TI "Gabon" OR AB "Gabon" OR SU "Gabonese Republic" OR TI "Gabonese Republic" OR AB "Gabonese Republic" OR SU "Gabonese" OR TI "Gabonese" OR AB "Gabonese" OR SU "Gambia" OR TI "Gambia" OR AB "Gambia" OR SU "Gambian" OR TI "Gambian" OR AB "Gambian" OR SU "Ghana" OR TI "Ghana" OR AB "Ghana" OR SU "Ghanaian" OR TI "Ghanaian" OR AB "Ghanaian" OR SU "Guinea" OR TI "Guinea" OR AB "Guinea" OR SU "Guinean" OR TI "Guinean" OR AB "Guinean" OR SU "Guinea-Bissau" OR TI "Guinea-Bissau" OR AB "Guinea-Bissau" OR SU "Guinea Bissau" OR TI "Guinea Bissau" OR AB "Guinea Bissau" OR SU "Guinea-Bissauan" OR TI "Guinea-Bissauan" OR AB "Guinea-Bissauan" OR SU "Kenya" OR TI "Kenya" OR AB "Kenya" OR SU "Kenyan" OR TI "Kenyan" OR AB "Kenyan" OR SU "Lesotho" OR TI "Lesotho" OR AB "Lesotho" OR SU "Basotho" OR TI "Basotho" OR AB "Basotho" OR SU “Sotho” OR TI “Sotho” OR AB “Sotho” OR SU "Liberia" OR TI "Liberia" OR AB "Liberia" OR SU "Liberian" OR TI "Liberian" OR AB "Liberian" OR SU "Madagascar" OR TI "Madagascar" OR AB "Madagascar" OR SU "Malagasy" OR TI "Malagasy" OR AB "Malagasy" OR SU "Malawi" OR TI "Malawi" OR AB "Malawi" OR SU "Malawian" OR TI "Malawian" OR AB "Malawian" OR SU "Mali" OR TI "Mali" OR AB "Mali" OR SU "Malian" OR TI "Malian" OR AB "Malian" OR SU "Mauritania" OR TI "Mauritania" OR AB "Mauritania" OR SU "Mauritanian" OR TI "Mauritanian" OR AB "Mauritanian" OR SU "Mayotte" OR TI "Mayotte" OR AB "Mayotte" OR SU "Mozambique" OR TI "Mozambique" OR AB "Mozambique" OR SU "Mozambican" OR TI "Mozambican" OR AB "Mozambican" OR SU "Namibia" OR TI "Namibia" OR AB "Namibia" OR SU "Southwest Africa" OR TI "Southwest Africa" OR AB "Southwest Africa" OR SU "South West Africa" OR TI "South West Africa" OR AB "South West Africa" OR SU "Namibian" OR TI "Namibian" OR AB "Namibian" OR SU "Niger" OR TI "Niger" OR AB "Niger" OR SU "Nigerien" OR TI "Nigerien" OR AB "Nigerien" OR SU "Nigeria" OR TI "Nigeria" OR AB "Nigeria" OR SU "Nigerian" OR TI "Nigerian" OR AB "Nigerian" OR SU "Rwanda" OR TI "Rwanda" OR AB "Rwanda" OR SU "Ruanda" OR TI "Ruanda" OR AB "Ruanda" OR SU "Rwandan" OR TI "Rwandan" OR AB "Rwandan" OR SU "Sao Tome and Principe" OR TI "Sao Tome and Principe" OR AB "Sao Tome and Principe" OR SU "Senegal" OR TI "Senegal" OR AB "Senegal" OR SU "Senegalese" OR TI "Senegalese" OR AB "Senegalese" OR SU "Sierra Leone" OR TI "Sierra Leone" OR AB "Sierra Leone" OR SU "Sierra Leonean" OR TI "Sierra Leonean" OR AB "Sierra Leonean" OR SU "Somalia" OR TI "Somalia" OR AB "Somalia" OR SU "Somalian" OR TI "Somalian" OR AB "Somalian" OR SU "South Africa" OR TI "South Africa" OR AB "South Africa" OR SU "South African" OR TI "South African" OR AB "South African" OR SU "South Sudan" OR TI "South Sudan" OR AB "South Sudan" OR SU "South Sudanese" OR TI "South Sudanese" OR AB "South Sudanese" OR SU "Sudan" OR TI "Sudan" OR AB "Sudan" OR SU "Sudanese" OR TI "Sudanese" OR AB "Sudanese" OR SU "Swaziland" OR TI "Swaziland" OR AB "Swaziland" OR SU "eSwatini" OR TI "eSwatini" OR AB "eSwatini" OR SU "Swazi" OR TI "Swazi" OR AB "Swazi" OR SU "Tanzania" OR TI "Tanzania" OR AB "Tanzania" OR SU "Tanganyika" OR TI "Tanganyika" OR AB "Tanganyika" OR SU "Zanzibar" OR TI "Zanzibar" OR AB "Zanzibar" OR SU "Tanzanian" OR TI "Tanzanian" OR AB "Tanzanian" OR SU "Togo" OR TI "Togo" OR AB "Togo" OR SU "Togolese" OR TI "Togolese" OR AB "Togolese" OR SU "Uganda" OR TI "Uganda" OR AB "Uganda" OR SU "Ugandan" OR TI "Ugandan" OR AB "Ugandan" OR SU "Zambia" OR TI "Zambia" OR AB "Zambia" OR SU "Zambian" OR TI "Zambian" OR AB "Zambian" OR SU "Zimbabwe" OR TI "Zimbabwe" OR AB "Zimbabwe" OR SU "Zimbabwean" OR TI "Zimbabwean" OR AB "Zimbabwean”)
